# A super pan-genomic landscape of rice

**DOI:** 10.1038/s41422-022-00685-z

**Published:** 2022-07-12

**Authors:** Lianguang Shang, Xiaoxia Li, Huiying He, Qiaoling Yuan, Yanni Song, Zhaoran Wei, Hai Lin, Min Hu, Fengli Zhao, Chao Zhang, Yuhua Li, Hongsheng Gao, Tianyi Wang, Xiangpei Liu, Hong Zhang, Ya Zhang, Shuaimin Cao, Xiaoman Yu, Bintao Zhang, Yong Zhang, Yiqing Tan, Mao Qin, Cheng Ai, Yingxue Yang, Bin Zhang, Zhiqiang Hu, Hongru Wang, Yang Lv, Yuexing Wang, Jie Ma, Quan Wang, Hongwei Lu, Zhe Wu, Shanlin Liu, Zongyi Sun, Hongliang Zhang, Longbiao Guo, Zichao Li, Yongfeng Zhou, Jiayang Li, Zuofeng Zhu, Guosheng Xiong, Jue Ruan, Qian Qian

**Affiliations:** 1 Shenzhen Branch, Guangdong Laboratory of Lingnan Modern Agriculture, Genome Analysis Laboratory of the Ministry of Agriculture and Rural Affairs, Agricultural Genomics Institute at Shenzhen, Chinese Academy of Agricultural Sciences, Shenzhen, Guangdong China; 2 State Key Laboratory for Agrobiotechnology, National Center for Evaluation of Agricultural Wild Plants (Rice), Department of Plant Genetics and Breeding, China Agricultural University, Beijing, China; 3 Academy for Advanced Interdisciplinary Studies, Plant Phenomics Research Center, Nanjing Agricultural University, Nanjing, Jiangsu China; 4 Department of Plant and Microbial Biology, University of California, Berkeley, Berkeley, CA USA; 5 Department of Integrative Biology, University of California, Berkeley, Berkeley, CA USA; 6 State Key Laboratory of Rice Biology, China National Rice Research Institute, Hangzhou, Zhejiang, China; 7 Key Laboratory of Molecular Design for Plant Cell Factory of Guangdong Higher Education Institutes, Institute of Plant and Food Science, Department of Biology, School of Life Sciences, Southern University of Science and Technology, Shenzhen, Guangdong, China; 8 Department of Entomology, College of Plant Protection, China Agricultural University, Beijing, China; 9 Grandomics Biosciences, Beijing, China; 10 State Key Laboratory of Agrobiotechnology/Beijing Key Laboratory of Crop Genetic Improvement, College of Agronomy and Biotechnology, China Agricultural University, Beijing, China; 11 State Key Laboratory of Plant Genomics, Institute of Genetics and Developmental Biology, Chinese Academy of Sciences, Beijing, China

**Keywords:** Structural variation, Comparative genomics

## Abstract

Pan-genomes from large natural populations can capture genetic diversity and reveal genomic complexity. Using de novo long-read assembly, we generated a graph-based super pan-genome of rice consisting of a 251-accession panel comprising both cultivated and wild species of Asian and African rice. Our pan-genome reveals extensive structural variations (SVs) and gene presence/absence variations. Additionally, our pan-genome enables the accurate identification of nucleotide-binding leucine-rich repeat genes and characterization of their inter- and intraspecific diversity. Moreover, we uncovered grain weight-associated SVs which specify traits by affecting the expression of their nearby genes. We characterized genetic variants associated with submergence tolerance, seed shattering and plant architecture and found independent selection for a common set of genes that drove adaptation and domestication in Asian and African rice. This super pan-genome facilitates pinpointing of lineage-specific haplotypes for trait-associated genes and provides insights into the evolutionary events that have shaped the genomic architecture of various rice species.

## Introduction

Rice is the most widely consumed crop.^1^ Improving rice productivity is essential to meet the growing demands of the ever-increasing world population.^2^ Two major cultivated species, Asian cultivated rice (*Oryza sativa*, *Os*) and African cultivated rice (*O. glaberrima*, *Og*), were domesticated independently. *Os* was domesticated from Asian wild rice (*O. rufipogon*, *Or*)^3^ and has two main types: *Geng* (*Os. japonica*, *Osj*) and *Xian* (*Os. indica*, *Osi*).^4^ *Osj* was domesticated as early as 9000 years ago,^5,6^ while *Osi* was formed later, with introgression of domestication alleles from *Osj*.^5^ About 3500 years ago, *Og* was domesticated from *O. barthii* (*Ob*), which diverged from *Or* approximately 600,000 years ago.^6,7^

The identification of a comprehensive set of genetic variations, including single nucleotide variations and structural variations (SVs), allows for investigation of the population structure and evolutionary dynamics of cultivated and wild rice, which has deepened understanding of the genetic basis for adaptation, domestication, and speciation.^4,7–10^ However, it should be noted that genetic variations are typically identified against a single reference genome; accordingly, DNA sequences that are absent or highly diverged from the reference genome are disregarded. Pan-genomes, which combine multiple genomes attempting to represent the entire set of genes for a species, can help overcome this issue of absent sequences. Four rice pan-genomes have been reported,^4,11–13^ including one constructed using short reads from 453 *Os* accessions,^4^ one iteratively assembled using short reads from 53 *Os* and 13 *Or* accessions,^12^ one assembled using long reads from 32 *Os* and 1 *Og* accessions^11^ and one assembled using long reads from 105 *Os* and 6 *Or* accessions.^13^ Notably, these pan-genomes primarily focused on *Os* accessions, with *Og*, *Or*, and *Ob* remaining underexplored.

Here, we integrated Oxford Nanopore Technology (ONT) long read data and Illumina short read data to generate high-quality assemblies of 251 rice genomes (202 *Os*, 28 *Or*, 11 *Og*, and 10 *Ob*). We constructed a graph-based pan-genome based on these assemblies and characterized its gene content. Finally, we conducted various analyses to illustrate that this fully annotated pan-genome is a valuable resource for understanding the genetic basis of trait variation, environmental adaptation, and domestication in rice.

## Results

### De novo assembly and annotation of 251 rice accessions

**Fig. 1:**
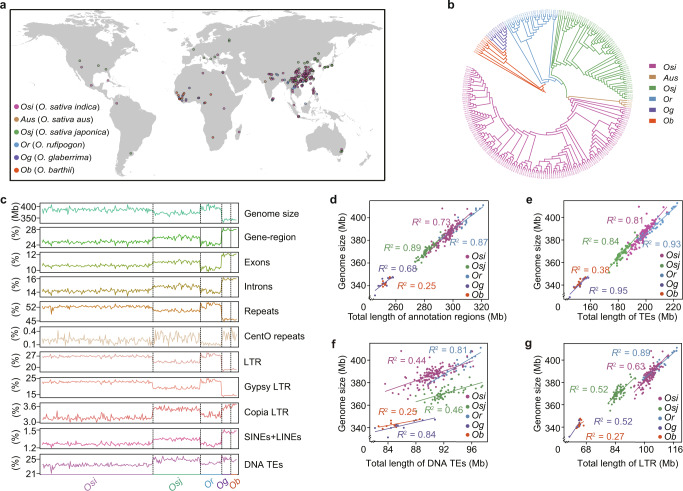
De novo assembly and annotation of the 251 accessions. **a** Geographic distribution of the 251 accessions examined in this study. Colored dots indicate the taxonomic classification of each accession. **b** Phylogeny of 251 accessions based on whole-genome SNPs. Accessions in different sub-populations are indicated by different colors. **c** Landscape of genome size and genomic elements across different sub-populations, including the percentage of gene-regions with different lengths, exons, introns, repeats, CentO repeats, LTR, Gypsy LTR, Copia LTR, SINEs + LINEs, and DNA TEs in genome. **d**–**g** Pearson correlation coefficients for comparisons between genome size and total length of annotation regions (**d**), the total length of TEs (**e**), the total length of DNA TEs (**f**) and total length of LTR (**g**) across different sub-populations. Colored dots and lines indicate data from each sub-population. *Osi*, *Aus*, *Osj*, *Or*, *Og*, and *Ob* respectively refer to *O. sativa indica*, *O. sativa aus*, *O. sativa japonica*, *O. rufipogon*, *O. glaberrima*, and *O. barthii*.

We selected 251 globally distributed rice accessions for their representativeness of the genetic and phenotypic diversity of global rice germplasm (Fig. 1a, b; Supplementary information Table S1a). In brief, we collected the *Os* accessions from a MiniCore collection, which was previously collected using a hierarchical sampling strategy from 50,526 rice varieties based on phenotypic and genetic variations.^14^ The *Or*, *Og* and *Ob* accessions used in the pan-genome were collected based on geographic diversity. In total, all accessions in the present study were collected from 44 countries (Supplementary information, Table S1a). The phylogenetic tree and admixture analysis based on whole-genome single nucleotide polymorphisms (SNPs) were used to remove the accessions that were not clustered together with accessions of the same reported sub-population classification (*Osi*, *Aus*, *Osj*, *Or*, *Og*, and *Ob* accessions) (Fig. 1b; Supplementary information Fig. S1a; Additional file 1 at https://zenodo.org/record/6602280). Since only 4 *Aus* accessions were collected in the present study, which is not enough for population analysis, *Aus* accessions were removed from the sub-population analysis. Finally, 135 *Osi*, 58 *Osj*, 26 *Or*, 11 *Og*, and 8 *Ob* were retained to represent the *Osi*, *Osj*, *Or*, *Og* and *Ob* sub-populations for population comparing analysis (Supplementary information, Table S1a). 251 accessions were used to analyze the genomic characteristics of rice and to compare the differences between Asian rice and African rice.

The 251 accessions were de novo assembled using WTDBG (version 2.5)^15^ with an average depth of 98 ± 24× using ONT long-read sequencing. The 251 accessions were also sequenced at an average depth of 64 ± 9× using short Illumina next-generation sequencing (NGS) to facilitate the correction during assembly. We ultimately generated 251 assemblies with average lengths of 386.4 ± 7.0 Mb, 370.6 ± 5.7 Mb, 394.6 ± 8.5 Mb, 342.2 ± 4.4 Mb and 342.7 ± 2.7 Mb for the *Osi*, *Osj*, *Or*, *Og* and *Ob* accessions, respectively (Supplementary information, Table S1b). The average contig N50 length of the 251 assemblies was 10.9 ± 3.7 Mb (Supplementary information, Table S1b).

We evaluated the assembly quality in the following four aspects, and found that: (1) the assembled genome size (9311, N22 and IR64) is comparable to that reported by a recent study^11^ (Supplementary information, Fig. S1b); (2) 97.7% ± 0.9% of NGS reads could be mapped to their corresponding assemblies, which is similar to the rate (98.0%) when mapping Nipponbare^16^ NGS reads to its genome (Supplementary information, Fig. S1c); (3) the completeness estimated by Benchmarking Universal Single-Copy Orthologs (BUSCO)^17^ was 96.4% ± 1.6%, which is comparable to the Nipponbare reference genome (97.6%) (Supplementary information, Fig. S1d and Table S1b); and (4) the analyses of collinearity against the Nipponbare reference genome (Additional file 2 at https://zenodo.org/record/6602280) and the high-throughput chromosome conformation capture (Hi-C) data from four assemblies (Supplementary information, Fig. S1e–h) indicate the high continuity and completeness of the 251 assemblies. These results suggested that the quality of all of our 251 genome assemblies were comparable to that achieved by the rice reference genome (Nipponbare) assembly.

To reveal the variation of genome size during rice domestication and speciation, we compared genome size among different sub-populations and observed a reduction of genome size during *Os* domestication, while genome size was comparable between *Og* and *Ob* (Fig. 1c; Supplementary information, Fig. S1i). Consistent with previous studies,^4^ *Osi* accessions (386.4 ± 7.0 Mb) had slightly larger genomes than *Osj* (370.6 ± 5.7 Mb). To understand the cause for differences in genome size among sub-populations, we annotated protein-coding genes for each genome and found that the species have a similar number of genes (34,974 ± 466), with slightly fewer genes in *Or* (34,863 ± 358, Supplementary information, Fig. S1j and Table S1c). In addition, we also annotated repeat sequences of the 251 assemblies using EDTA.^18^ The average sequence length of transposable elements (TEs) per assembly was 191.9 Mb, accounting for an average of 50.5% of the total assembly length (Supplementary information, Table S1d). The observed variations in genome size can be primarily explained by the number of TEs (Fig. 1d; Supplementary information, Fig. S1k, l), particularly by long terminal repeats (LTRs) (Fig. 1e–g; Supplementary information, Table S1d).

### A super pan-genome of cultivated rice and wild rice

**Fig. 2:**
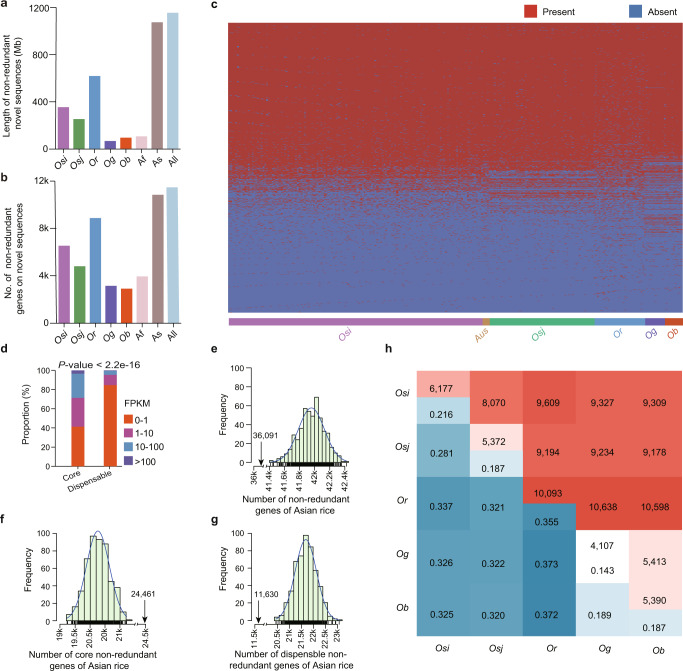
Super pan-genome of 251 wild and cultivated Asian and African *Oryza* accessions. **a** Total length of non-redundant novel sequences detected from the super pan-genome. Non-redundant novel sequences mean sequences that were absent in the Nipponbare reference genome and do not show redundancy across genomes. **b** Number of non-redundant genes in the non-redundant novel sequences. **c** The Landscape of PAVs for non-redundant genes across the 251 accessions. Each row indicates a non-redundant gene and each column indicates an accession. If the member of the non-redundant gene was present in an accession, it was colored in red; otherwise, it was colored in blue. Non-redundant genes were sorted by their occurrence. **d** Gene expression landscape of core and dispensable non-redundant genes. A Wilcoxon test was applied to the FPKM values. **e**–**g** Bootstrapping of all (**e**), core (**f**), and dispensable genes (**g**) in 11 *Os* and 10 *Or*. 11 *Os* and 10 *Or* were randomly selected 500 times from 230 Asian rice. The black arrows indicate the numbers of all, core and dispensable genes in 21 African rice (11 *Og* and 10 *Ob*), respectively. Non-redundant genes present in ≥ 95% accessions are defined as core non-redundant genes, and the rest of non-redundant genes are dispensable. **h** The average ratio (numbers) of different non-redundant genes when comparing two accessions. *Os*, *Osi*, *Osj*, *Or*, *Og, Ob*, Af, As and All respectively refer to *O. sativa*, *O. sativa indica*, *O. sativa japonica*, *O. rufipogon*, *O. glaberrima, O. barthii*, African rice, Asian rice and the 251 accessions.

A super pan-genome is a pan-genome constructed from the genomes of different species within a genus.^9^ Unifying genomic features by the super pan-genome enables functional and evolutionary studies of genes across different species or populations. We constructed a graph-based pan-genome incorporating the polymorphisms in orthologous regions across high-quality assemblies of Asian and African cultivated rice and wild rice accessions. This pan-genome consisted of 1.52 Gb non-redundant DNA sequences across genomes, including 1.15 Gb sequences absent in the Nipponbare reference genome (Fig. 2a, b). Surprisingly, the 1.15 Gb sequences were mainly contributed by *Or*, which is evolutionarily closer to Nipponbare than to *Og* or *Ob* (Fig. 2a), possibly because of the higher genetic diversity of *Or* compared to *Og* and *Ob*^19^ and the smaller number of African rice genomes used in this study compared to the number of *Or* genomes.

We clustered the genes in all assemblies of the pan-genome using OrthoFinder (https://github.com/davidemms/OrthoFinder). Each group of clustered genes (i.e., orthogroup) was defined as a non-redundant gene. In total, 51,359 non-redundant genes were annotated for the pan-genome, including 21,888 core genes (i.e., those present in ≥ 95% of the accessions) and 29,471 dispensable genes (Fig. 2c). The core genes are expressed at a higher level than the dispensable genes (*P* < 2.2e−16; Fig. 2d). In the super pan-genome, 34,001 genes were present in both the *Or* and *Ob* accessions. In addition, 10,101 genes were present only in Asian rice and 1259 genes were present only in African rice (Supplementary information, Fig. S2a). To estimate the representativeness of these accessions, the total number of non-redundant genes present in a population was estimated by computing the change in the number of non-redundant genes each time a new genome was added. After randomizing the order of rice accessions 500 times, our simulation analysis suggested the total number of Asian rice genes approached a plateau (Supplementary information, Fig. S2b) and the number of non-private genes (non-private genes are defined as non-redundant genes present in at least two accessions) in African rice was close to a plateau (Supplementary information, Fig. S2c). To reduce the effect of the unbalanced sample size of Asian (*n* = 230) and African accessions (*n* = 21) on comparing Asian and African rice characteristics, we down-sampled the Asian accessions to 21 accessions, and our data indicated that Asian rice has a larger gene set than African rice, with fewer core genes and more dispensable genes than African rice (Fig. 2e–g). The evolutionary divergence between the *Osi* and *Osj* genomes of *O. sativa* long predates the domestication of *O. sativa* from *Or*. This means that within the single *Or* species there are at least two highly diverged genome types.^5,6^ To estimate gene variations within and between sub-populations, we calculated the average numbers of genes that are different between two accessions and found that *Or* likely has the highest intra-species diversity, with an average of 35.5% of genes showing presence/absence variations (PAVs) between any two randomly selected *Or* accessions, a level that is comparable to the difference between a typical Asian accession and an African accession (33.25%) (Fig. 2h). The ancient genome divergence between the *Osi* and *Osj* genomes may account for the high intra-species genomic diversity for *Or*.

Asian accessions had larger variations than African rice accessions as the difference in gene content between *Or* and *Osi*/*Osj* is greater than that between *Ob* and *Og* (Fig. 2h). The gene PAVs could then be used to clearly distinguish the sub-populations (Supplementary information, Fig. S2d). We found considerable variations in the functional genes among sub-populations. We also analyzed and verified PAVs of some functional genes (Supplementary information, Fig. S2e–i). For example, the *OsSh1* gene, which was previously reported to cause a shattering-resistant phenotype when its expression is down-regulated,^20^ was specifically absent in the non-shattering *Og*. Another gene, *OsLCT1*, that encodes a protein known to effectively decrease the translocation and accumulation of cadmium (Cd) into grains when overexpressed,^21^ was present in most *Osj* accessions but absent in all the *Osi* accessions, consistent with the observation that *Osj* accessions generally show lower levels of Cd than *Osi* accessions^22^ (Supplementary information, Fig. S2e–i). The lineage-specific distribution of PAVs of functionally characterized genes indicates that discerning haplotypes of functional genes in different species has the potential to identify lineage-specific elite genes which could be introduced into other sub-populations for rice improvement.

To provide access and tools for exploring these genomic resources, we developed the Rice Super Pan-genome Information Resource Database (http://www.ricesuperpir.com/). A reference-free whole-genome multiple sequence alignment for the 251 accessions was performed with Cactus software.^23^ The resulting alignment can be visualized using any assembly as the reference with this database. The database also integrated the SVs, gene annotations, TE annotations, pan-genome graph, and BLAST tools.

### Construction and characterization of a rice pan-NLRome

A family of highly diverse genes known as the nucleotide-binding leucine-rich repeat receptors (NBS-LRRs, NLRs) function in plant immunity by specifically recognizing pathogen effectors.^24^ A species-wide inventory of NLRs should serve as a valuable resource for future breeding efforts toward disease resistance.^7^ The NLRome is an important part of the rice pan-genome. The pan-NLRome can obtain NLR genotypes and allelic or orthologous relationships between accessions or species. It can potentially solve the problem of low efficiency of traditional linkage or association analysis for NLRs and display more directly large SV and copy number variation (CNV) with NLRs.^25^ It has been shown in *Arabidopsis* that there are large differences in NLRs between accessions, and it is still impossible to determine the true degree of NLR diversity by fewer species.^26^ Therefore, we assembled high-quality rice genomes using long reads data to analyze NLR diversity.^26,27^

**Fig. 3:**
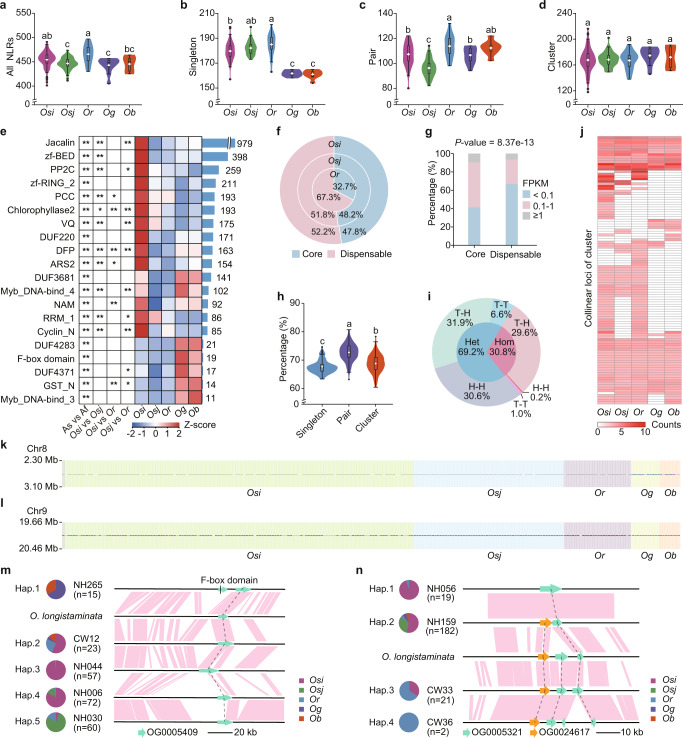
Characterization of NLRs in super pan-genome. **a**–**d** Gene numbers across different sub-populations: total number of all NLRs (**a**), singleton NLRs (**b**), paired NLRs (**c**), and clustered NLRs (**d**). The white dots indicate the mean values. The lowercase letters in the figure reflect the levels of statistical significance assessed with the Kruskal-Wallis tests (with Bonferroni’s multiple comparison post hoc tests). **e** Summary of integrated domain in NLRs. The heatmap indicates domains’ frequencies (Z-score transformed) among the sub-populations. We used the Wilcoxon test with FDR adjustment to infer the enrichment of a specific domain in a given sub-population. * adjusted *P* < 0.05, and ** adjusted *P* < 0.01. The barplot indicates the total number of integrated domain identified in all accessions. The figure only shows integrated domain observed over 10 times and with significant differences between Asian and African accessions. The results for all integrated domain are shown in Supplementary information, Table S2b. **f** The percentage of core or dispensable non-redundant NLRs in the Asian sub-population, including *Osi*, *Osj*, and *Or*. **g** Expression of core and dispensable non-redundant NLRs. A Wilcoxon test was applied to analyze the raw expression values. **h** The percentage of singleton, paired, and clustered NLRs among the core NLRs. The white dots indicate the mean values and the lowercase letters reflect the significance. Kruskal-Wallis tests (with Bonferroni’s multiple comparison post hoc tests) was used for the statistical significance analysis. **i** Combination pattern of paired NLRs. The inner ring represents the homogeneous rate (pink) and the heterogeneous rate (blue) of pair formation. The outer ring indicates the gene arrangements. H-H, T-T, and T-H respectively refer to the arrangements head-to-head, tail-to-tail, and tail-to-head. **j** The average number (the number of NLRs contained in the cluster) of collinearity loci in different sub-populations. **k**, **l** Example collinearity loci of singleton, paired, and clustered NLRs on Chr8 (**k**) and Chr9 (**l**). Gray, blue, and red dots indicate singleton, paired, and clustered NLRs, respectively. **m**, **n** The allelic variation among the sub-populations of the collinearity loci Chr8: 2,778,922−2,890,239 (**m**), Chr9: 20,154,563−20,167,795 (**n**). As, Af, *Osi*, *Osj*, *Or*, *Og* and *Ob* refer to Asian rice, African rice, *O*. *sativa indica*, *O*. *sativa japonica*, *O*. *rufipogon*, *O*. *glaberrima*, and *O*. *barthii*, respectively.

NLRs tend to cluster together in the genome and contribute to plant defense,^7,24^ thus based on the distribution of NLRs in each accession, we classified the NLRs as singletons, pairs or clusters. We observed that the number of each type varies in the sub-populations. The Asian rice accessions contained more singleton NLRs than their African siblings' species (Fig. 3a–d; Supplementary information, Table S2a). Moreover, the cultivated Asian rice accessions contained fewer paired NLRs than their wild progenitors (Fig. 3c).

Another sign of NLRome diversity across sub-populations is the change in different integrated domain architectures.^24,28^ These domains may relate to proteins that are repeatedly affected by pathogens, and their recognition provides targets that lead to the identification of new pathogen effectors.^29^ Among the total of 113,687 NLRs of all accessions, 10,154 have at least one non-canonical NLR domain (Fig. 3e; Supplementary information, Table S2b). The domains of NLRs in different sub-populations show different patterns with the singleton, paired, and clustered NLRs containing different domains (Supplementary information, Fig. S3a).

To better compare NLRs in multiple genomes, we constructed a rice pan-NLRome with all NLRs from 251 accessions. Finally, we got 496 non-redundant NLRs (Supplementary information, Table S2c). We compared the characteristics of non-redundant NLRs among sub-populations. The core or dispensable non-redundant NLRs were determined based on their distribution. Among the Asian rice accessions, wild accessions contained a higher proportion of dispensable NLRs than the cultivated accessions (Fig. 3f). More than 80% of NLRs were found to be shared between *Or* and *O*b (Supplementary information, Fig. S3b), suggesting that most NLRs in cultivated rice were retained from wild rice. All of these dispensable genes can better maintain the diversity of NLRs, providing the opportunity to potentially analyze the diversity of all or at least most of the NLR loci. However, there were no more dispensable NLRs in *Ob* than *Og* (Supplementary information, Fig. S3c). It is generally believed that NLRs are expressed at low levels under normal conditions.^30^ We interestingly found that a higher portion of the core NLRs showed expression as compared with the dispensable NLRs (*P*-value = 8.4e−13; FPKM > 0.1; Fig. 3g), whereas there are more expressed genes in *Or* whose core NLRs were in a lower number (Supplementary information, Fig. S3d). This phenomenon indicates that some dispensable NLRs in *Or* may have not been preserved during evolution. We also found that paired and clustered NLRs were more likely to be core NLRs than singleton ones (Fig. 3h), which suggests that these pairs or clusters of NLRs might be more conserved and retained in the course of evolution.

In addition, the genomic arrangement of NLRs further contributes to the diversity of this gene family.^7^ Since paired NLRs usually act as helpers and sensors to function together,^31^ we investigated the arrangement of paired NLRs in the accessions. Interestingly, the NLRs in homologous pairs (with the same non-redundant genes) were almost exclusively found in tail-to-head (T-H) arrangement, whereas those in heterologous pairs (with different non-redundant genes) were also found in either head-to-head (H-H) or tail-to-tail (T-T) arrangement (Fig. 3i). This distinction in gene orientation may indicate different evolutionary origins of the homologous and heterologous NLR pairs. Although we do not understand the effects of these arrangements on the function of NLRs, our study confirms that the arrangement of NLRs in rice populations is non-random.

To further understand the arrangement and diversity of NLRs, it is necessary to identify the NLRs’ collinear loci across accessions. A pan-genome graph provides a feasible solution.^26^ We identified the NLRs in the pan-genome by mapping them to the pan-genome graph and inferring the NLR loci through the pan-genome bubbles overlapped with NLRs in the corresponding assembly. Although the number of clustered NLRs was similar across sub-populations (Fig. 3d; Supplementary information, Table S2a), we found that the number of NLRs at each given locus could diverge dramatically across sub-population (Fig. 3j). We also found evidence of extensive PAVs of some loci among rice sub-populations: some NLR cluster loci found in Asian rice accessions were absent in the syntenic regions of African rice genomes (Supplementary information, Fig. S3e–p). For example, a heterologous paired NLR locus on Chromosome 8 of African rice was syntenic to a singleton NLR among Asian rice accessions. At this locus, the African rice orthologs contained either a conserved integrated domain homologous to *Pi36*^32^ or a lineage-specific F-box domain, neither of which was found in the orthologous singleton NLR in Asian rice genomes (Fig. 3k, m). Similarly, another NLR locus corresponding to a three-gene NLR cluster on Chromosome 9 of Asian rice has at least three NLRs among African rice genomes, which means one more copy is present at this locus in many African rice (Fig. 3l, n).

### Pan-SV identification and characterization

To survey the landscape of structural variation in rice, high-quality ONT sequencing reads from the 251 accessions were aligned to the Nipponbare^16^ genome using minimap2^33^ and NGMLR,^34^ and SVs were called using Sniffles.^34^ We called a total of 193,880 SVs (including deletions, DELs; insertions, INSs; inversions, INVs; translocations, TRAs; and duplications, DUPs) (Supplementary information, Table S3a) against the Nipponbare^16^ reference genome. A typical accession has 2660 to 32,097 SVs, depending on its evolutionary distance with the reference genome (Supplementary information, Fig. S4a and Table S3b). To quantitatively estimate the accuracy of SV calling, we manually examined 500 randomly selected SVs by visualizing the corresponding long-read alignment through an Integrative Genomics Viewer Browser. The SV calling accuracy was estimated to be 95.8% (Supplementary information, Table S3c). To validate large SVs, we performed Hi-C sequencing of four accessions (NH229, NH231, NH265 and NH286) and mapped the Hi-C paired reads to the corresponding genome assemblies using a chromatin interaction heatmap at 5 kb resolution with Juice (Supplementary information, Fig. S4b–e and Table S3c).

To assess whether our current population has reached SV saturation, and to see how many SV frequencies our Asian and African rice pan-genome covers, we then compared the SV content among rice species, and our simulation analysis suggested that the number of non-private SVs (non-private SVs are defined as SVs present in at least two rice accessions) in Asian rice was close to a plateau and the number of non-private SVs in African rice was close to a plateau (Supplementary information, Fig. S4f, g). The majority of SVs are relatively short (66.5% are less than 1 kb in length) (Supplementary information, Fig. S4h, i) and relatively rare (73.2% of SVs have a minor allele frequency < 0.05 and more than 65.9% of SVs were identified in at least two accessions) (Supplementary information, Fig. S4i). We identified 2,811 putative SV hotspots across the different sub-populations, with enrichment on the long arm of Chromosome 11 in *Os*, but not in *Or*, *Og*, or *Ob* (Supplementary information, Fig. S4j), and the difference of SV numbers in each window between variants and simulated variants was significantly different using Wilcoxon test (*P* < 0.01). This hotspot overlapped with many NLR genes (Supplementary information, Fig. S3e–p) and has been functionally implicated in defense responses against bacteria.^11,35^

### SVs can affect agronomic traits by altering gene expression

**Fig. 4:**
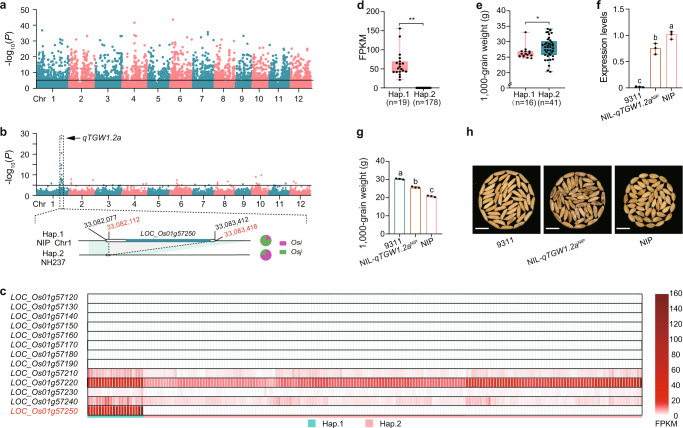
SVs can affect agronomic traits by altering gene expression. **a** Manhattan plot of the associations of SVs from the pan-SV dataset and gene expression levels. Only cis results (associations of SVs and their nearby genes (within 2 kb)) were selected from the results of associations of all filtered SVs with all filtered genes and displayed. **b** Manhattan plot of the associations between the pan-SVs and expression levels of 13 candidate genes of QTL *qTGW1.2a*. A 1.3 kb DEL was strongly associated with the expression of *LOC_Os01g57250*. **c** Expression levels of the 13 candidate genes in QTL *qTGW1.2a* in leaves of each accession. The FPKM value is represented by different colors, with white indicating low and red indicating high values. Hap.1 and Hap.2 indicate the presence/absence of the 1.3 kb SV in *LOC_Os01g57250*. **d**, **e** FPKM of *LOC_Os01g57250* (**d**) and TGW (**e**) of accessions with (Hap.2) or without (Hap.1) the SV in *LOC_Os01g57250*. Significance was tested by Wilcoxon tests (**d**, **e**). *P < 0.05, and **P < 0.01. **f** Expression levels of *LOC_Os01g57250* in young leaves (*n* = 3) investigated by qPCR. The letters indicate statistical significance levels from one-way ANOVA with Tukey's test (*P* < 0.05). **g**, **h** Comparison of TGW among NIP, 9311, and NIL-*qTGW1.2a*^NIP^ (*n* = 3). Scale bars, 1 cm. The letters indicate statistical significance levels from one-way ANOVA with Tukey's test (*P* < 0.05).

The expression of genes can be altered by nearby SVs due to their interruptions in gene or regulatory sequences. For example, a 520 bp DEL in the promoter of *DNR1* in *Osi* accessions such as HJX74 reduced *DNR1* transcript levels and improved nitrogen uptake rates in *Osi* compared to *Osj*.^36^ To explore the relationship between SVs and gene expression, we cataloged the SVs across our rice pan-genome and found that 35.4% of genes were flanked by SVs (overlapping with coding regions or putative regulatory elements) (Supplementary information, Fig. S4k). To discover SVs affecting gene expression, we associated SVs with the gene expression levels among *Os* accessions and identified expression quantitative trait loci (eQTLs) (Fig. 4a). These eQTLs included some candidate genes that may be responsible for important agronomic traits, such as grain size. For example, the expression of a known grain-weight gene *HGW* (*LOC_Os06g06530*)^37^ was significantly associated with a 127 bp INS upstream of the gene (Supplementary information, Fig. S5a–e). The accessions with the INS had reduced thousand grain weight (TGW) compared to those without the INS (Supplementary information, Fig. S5f). Likewise, the down-regulation of a nicotinate phosphoribosyltransferase gene *OsNaPRT1* (*LOC_Os03g62110*)^38^ was related to a downstream DEL (Supplementary information, Fig. S5g–k). The accessions with the DEL (Hap.2) showed lower TGW than those without the DEL (Hap.1) (Supplementary information, Fig. S5l). Another eQTL was found near the QTL *qTGW1.2a*,^39^ which was related to TGW (Fig. 4b), and was fine-mapped to a 77.5 kb region on Chromosome 1 containing 13 candidate genes. Among these genes, *LOC_Os01g57250* was covered by a 1.3 kb SV (Fig. 4b). The expression of *LOC_Os01g57250* was detected in Hap.1 accessions with the 1.3 kb sequence but not in Hap.2 accessions lacking the 1.3 kb sequence (Fig. 4c, d). The haplotypes of *LOC_Os01g57250* showed a significant difference in TGW in the *Osj* accessions, with Hap.1 associated with lower TGW, indicating that *LOC_Os01g57250* negatively regulated TGW (Fig. 4e). Consistently, a near-isogenic line in the 9311 genetic background with a *qTGW1.2a* region introgressed from Nipponbare had higher *LOC_Os01g57250* expression levels and a lower TGW than 9311 (Fig. 4f–h). These results suggest that *LOC_Os01g57250* is a negative regulator of TGW and is likely the causal gene underlying the QTL *qTGW1.2a*.

### SVs associate with agronomic traits

**Fig. 5:**
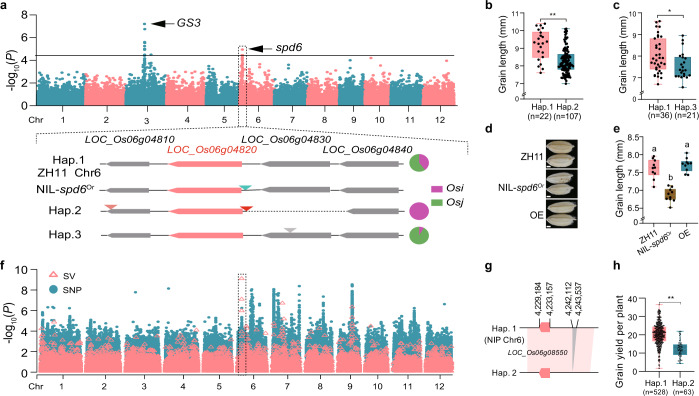
GWAS analysis using SVs. **a** GWAS for grain length using the pan-SV dataset. Complex SVs occurred within the QTL *spd6*, exhibiting three major haplotypes. **b**, **c** Comparison of grain length in Hap.1 and Hap.2 of *Osi* (**b**) and Hap.1 and Hap.3 of *Osj* (**c**). *Osi* and *Osj* respectively refer to *O. sativa indica* and *O. sativa japonica*. Significance was tested by two-tailed *t*-test (**b**) and Wilcoxon tests (**c**). *P < 0.05, and **P < 0.01. **d**, **e** Comparison of grain length of ZH11, NIL-*spd6*^*Or*^ (near-isogenic lines, NIL), and a transgenic plant with cDNA of *LOC_Os06g0482*0 from ZH11 over-expression in the NIL-*spd6*^*Or*^ background (*n* = 3). Scale bars, 1 mm. The letters indicate statistical significance levels from one-way ANOVA with Tukey's test (*P* <  0.05) (**e**). **f** GWAS for grain yield per plant using SVs genotyped based on the variation graph from previously published NGS data. The locus for grain yield on Chromosome 6 could only be identified by SVs but not by SNPs. **g** The most significant SV was a 1.4 kb DEL at the promoter of *LOC_Os06g08550*. **h** Comparison of grain yield per plant between two haplotypes of *LOC_Os06g08550*. The statistical significance was inferred by the Wilcoxon tests. ** *P* < 0.01.

The SVs that determine agronomic traits in rice have been recognized in recent years. For example, the 1,116 bp DEL in the *DTH8* gene (*LOC_Os08g07740*)^40^ and the 17.1 kb CNV of *GL7* (*LOC_Os07g41200*)^41^ were reported to affect rice heading date and promote grain length, respectively. Discerning haplotypes of SVs across sub-populations can also facilitate the identification of beneficial haplotypes for rice improvement. We generated a phylogenetic tree based on our SV dataset, which clearly separated the sub-populations, and showed a structure that mirrored the SNP-based rice phylogeny (Fig. 1b; Supplementary information, Fig. S5m). Thus, we next analyzed the distribution of the functionally characterized SVs among the sub-populations. The result showed that the DEL in *DTH8* is only present in *Osi* accessions, while the CNV of *GL7* is only found in *Osj* accessions (Supplementary information, Fig. S5n). Other than the known functional SVs, we also found SVs that were present specifically in African rice accessions, such as a 1.8 kb INS in the first exon of *RFT1* (Supplementary information, Fig. S5n). To demonstrate how the pan-SVs can be used to facilitate the identification of trait-associated SVs, we conducted a genome-wide association analysis of grain length in *Os*. In addition to *GS3*, we identified a locus close to *spd6*, a previously identified QTL for panicle length, plant height, and grain size (Fig. 5a). *spd6* was identified in recombinant inbred lines derived from a cross between the *Or* accession Y2 and the *Osi* accession Teqing and contains four candidate genes.^42^ To determine the functional gene for *spd6*, we analyzed the haplotypes of the locus and found that variations between Hap.1 and Hap.2 in *Osi* and between Hap.1 and Hap.3 in *Osj* were associated with differences in grain length (Fig. 5a–c), thus narrowing down the candidate genes to *LOC_Os06g04820* and *LOC_Os06g04830*. To confirm the causal gene, we then sequenced the near-isogenic line NIL-*spd6*^*or*^, in the *Osj* cv. ZH11 background containing the *spd6* segment derived from *Or* accession Y2. Sequence analysis revealed that a 4 kb INS occurred at 53 bp upstream of *LOC_Os06g04820* in NIL-*spd6*^*or*^ (Fig. 5a). These results allowed us to infer that *LOC_Os06g04820* is the causal gene for *spd6*. Further, to verify the function of *LOC_Os06g04820*, the *LOC_Os06g04820* cDNA from ZH11 was introduced into NIL-*spd6*^*Or*^ and was found to rescue the grain length phenotype (Fig. 5d, e), strongly supporting that the 4 kb INS disrupts *LOC_Os06g04820* function and thus decreases the grain length in NIL-*spd6*^*Or*^.

To date, population studies have relied mostly on high-throughput short-read DNA sequencing technologies. To facilitate the identification of the SVs from short-read data and utilize the corresponding phenotypic data, we constructed a variation graph based on the Nipponbare reference genome and the pan-SV dataset. We called SVs by mapping published short-read sequence data from 605 *Os* rice accessions to the variation graph (Supplementary information, Table S4).^43^ Then we performed a genome-wide association study (GWAS) for grain yield with the identified SVs. This identified a grain yield-associated SV (a 1.4 kb DEL compared to Nipponbare) near *OsNPY2* (*LOC_Os06g08550*).^44^ Notably, these signals could not be detected by SNPs against the Nipponbare reference genome (Fig. 5f–h).^16^ These results emphasize one major advantage of using a pan-genome to identify trait-associated genetic variations in rice.

### Adaptation in Asian and African rice

**Fig. 6:**
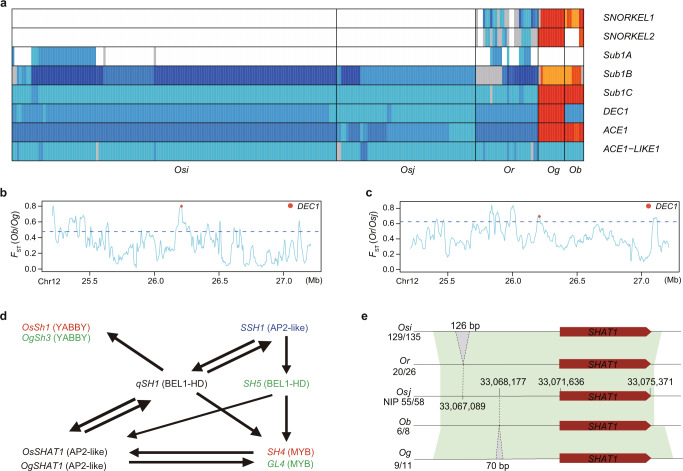
Submergence adaption and domestication shattering of Asian and African rice. **a** Genotypes of genes regulating submergence responses in each accession across sub-populations. Each column was an accession. Different colored boxes indicate different haplotypes. The blank boxes indicate gene absence in the corresponding accession. A gray box indicates the haplotype only present in the corresponding accession; boxes with other colors indicate haplotypes present in more than one accession. **b**, **c** *F*_ST_ values (computed based on SNPs at 20 kb resolution) between *Ob* and *Og* (**b**), and between *Or* and *Osj* (**c**) in a 2 Mb genomic region of *DEC1* gene. **d** Genetic network that regulates shattering in rice. Blue, red, or green indicate genes domesticated in *Osi*, *Os*, or *Og*, respectively. **e** Haplotypes of the *SHAT1* gene*. Os, Osi, Osj, Or, Og* and *Ob* respectively refer to *O. sativa, O. sativa indica, O. sativa japonica, O. rufipogon, O. glaberrima* and *O. barthii*.

To adapt the adverse environments on Earth, including severe and unfavorable environmental conditions for living organisms, plants have evolved many biological functions.^45–47^ Dissecting the genetic basis underlying local environmental adaptation is important to understand the evolution of rice. Flooding is a severe abiotic stress that imposes strong selection on rice.^48^ Deepwater rice accessions can survive during long-term submergence and are grown in flood-prone environments in South Asia and West Africa.^48^ The genetic basis underlying adaptation for submergence has been well characterized in Asian rice,^49–52^ but is not well known in African rice. To figure out whether African rice species have developed a different genetic mechanism or use a strategy similar to that in Asian rice adapted to survive in flood-prone environments, we explored the genotypes of several genes reported to be involved in submergence such as *Sub1A/B/C*, *SNORKEL1/2*, *DEC1*, *ACE1* and *ACE1-LIKE1* using our pan-genome (Fig. 6a). We found that *Sub1A*, a gene previously reported to positively regulate the resistance of rice to submergence in a “submergence quiescent strategy”,^52^ was absent from *Ob* and *Og* accessions, whereas *SNORKEL1*/*2* and *ACE1* were present in both *Ob* and *Og* (Fig. 6a; Supplementary information, Table S5). *SNORKEL1*/*2*^49^ and *ACE1* are positive regulators of submergence-induced internode elongation,^51^ while *DEC1* is a negative regulator of this trait.^51^ These findings indicate that submergence-induced internode elongation has been employed as a major adaptive mechanism to escape submergence stress in both Asian and African rice. By the above haplotype analysis, we newly found that *OgDEC1* has a 54 bp in-frame INS, which may affect its function (Supplementary information, Table S5). To determine whether the observed differential distribution of genotypes of these submergence tolerance genes among sub-populations was due to selection, we performed *F*_ST_ analysis. The results show selection on *SNORKEL2* orthologues (*F*_ST_ = 0.75, rank: 2.25%) and *DEC1* (*F*_ST_ = 0.79, rank: 0.07%) in African rice and suggest that independent selection on these submergence tolerance genes may have contributed to the adaptation of *Og* to survive in flood-prone environments (Fig. 6a–c).

### Domestication in Asian and African rice

Cultivated rice differs phenotypically from wild rice. The genes contributing to these domestication phenotypes can be inferred from highly diverged outliers between wild and cultivated rice. We compared wild rice to cultivated rice by estimating genomic divergence (both SVs and SNPs) using metrics including *F*_ST_ (Supplementary information, Fig. S6a–h) and *D*_*xy*_ (Supplementary information, Fig. S6i–n) in both Asian and African rice (Additional file 3 at https://zenodo.org/record/6602280). *Os* and *Og* are independently domesticated rice species that display parallel genetic changes, such as a distinct loss-of-function in the same gene that caused parallel evolutionary changes in phenotypes in both species. Loss of shattering is a primary domestication trait in both *Os* and *Og*.^53–58^ Multiple loss of function events in orthologous genes, such as *qSH1*, *OgGL4*/*Ossh4* and *Ogsh3*/*OsSh1*, are known to have been independently domesticated in Asian and African rice. Our data suggested that the parallel selection for loss of shattering in Asian and African rice was driven by the independent selection of variations in different components of a genetic network (Fig. 6d; Supplementary information, Fig. S6); in each wild rice to cultivated rice comparison, genes related to loss of shattering were identified in highly divergent regions. In addition, we found an orthologue of *SHATTERING ABORTION1* (*SHAT1*)^58^ in a region that diverged between *Ob* and *Og* (Supplementary information, Fig. S6k), and identified a ~126 bp insertion 4.5 kb upstream of *SHAT1* in *Osi* comparing to *Or* and a ~70 bp insertion 3.5 kb upstream of *SHAT1* in *Og* compared to *Ob* in the *SHAT1* locus (Fig. 6e). These results indicate that the selection of different variants in *SHAT1* orthologues may have contributed to the domestication of shattering traits in Asian and African rice.

**Fig. 7:**
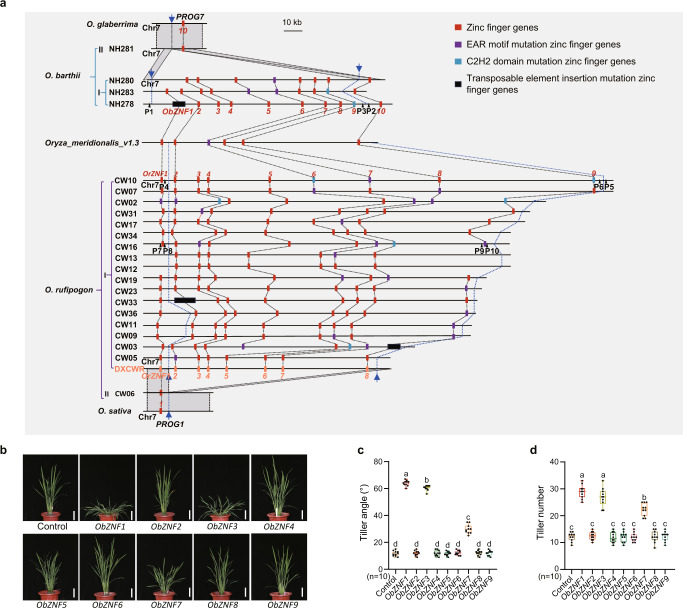
Structural variations in the *RPAD* locus in Asian and African rice. **a** SVs in the *RPAD* locus. The red, purple, blue, and black boxes respectively represent zinc-finger (ZNF) genes, EAR motif mutated ZNFs, C2H2 domain mutated ZNFs, and ZNFs with TE insertions. The dotted black lines indicate orthologous relationships. Gray regions indicate collinear sequences. Blue arrows and the corresponding dashed lines indicate deletion sites. Black triangles and P1−P10 indicate the positions and names of the primers used for validation of variations in *RPAD* locus. **b** Plant architecture of the control (GIL28) and the transgenic plants of *ObZNF1–ObZNF9*. Scale bars, 10 cm. **c**, **d** Comparison of the tiller angle (**c**) and tiller number (**d**) between the control and transgenic plants transformed with the indicated *ObZNFs* (*n* = 10). The letters indicate statistical significance assessed using one-way ANOVA with Tukey's test (*P* <  0.05) (**c**, **d**).

Rice domestication also involved a transition from prostrate to erect growth, and this is known to have been mediated by *PROG1*^59,60^ in Asian rice and by *PROG7*^61^ in African rice. *OsPROG1* and *OgPROG7* are syntenic and orthologs. They both encode zinc-finger transcription factors.^61^ We found that Hap.1 of *PROG1* was fixed in *Os*, while Hap.1 of *PROG7* was fixed in *Og* (Supplementary information, Fig. S7a, b). A ~110 kb deletion of the *RICE PLANT ARCHITECTURE DOMESTICATION* (*RPAD*) locus, which contains an array of zinc finger genes (ZNFs) including *PROG1*, was previously suggested to have affected plant architecture domestication in Asian rice,^60^ which highlights known impacts of large-size SVs in crop domestication. A similar but independent large deletion also occurred, which was at a slightly different location within the *RPAD* locus, in African rice.^60^ We found that deletions of the *RPAD* locus in both *Or* and *Os* accessions overlapped with deletions existing in both *Ob* and *Og* accessions (Fig. 7a; Supplementary information, Fig. S7c–e). Among the *OrZNFs* at the *OrRPAD* locus, *OrZNF5*, *OrZNF7*, and *OrZNF8* are known to regulate plant architecture.^60^ However, the functional zinc finger genes except *PROG7* in the *RPAD* locus of African wild rice are still unclear. Therefore, we constructed the complementary constructs for *ObZNF1*−*ObZNF9* in the *RPAD* locus of African wild rice and transferred the nine constructs separately into the introgression line GIL28. Transgenic results indicated that transgenic plants of *ObZNF1*, *ObZNF3*, *ObZNF7* showed larger tiller angles and increased tiller numbers compared with the control plants (Fig. 7b–d). We conclude that among the *ObZNFs* at the *ObRPAD* locus, only the *ObZNF1*, *ObZNF3*, and *ObZNF7* regulate plant architecture in African rice. *ObZNF1* is the ortholog of *OrPROG1*, while *ObZNF10* is orthologous to *OrZNF8*. These results showed that the selection of independent deletions has resulted in retaining *OrZNF1*/*OsPROG1* in Asian rice and *ObZNF10*/*OgPROG7* in African rice (Fig. 7a). The *ObZNF10*/*OgPROG7* is homologous to *OrZNF1*/*OsPROG1* (Supplementary information, Fig. S7f, g). In summary, the parallel domestication of plant architecture in Asian and African rice has been driven by the independent selection of both the large-size deletions and the functions of distinct members of an array of zinc finger genes at a single, orthologous genomic region.

## Discussion

Asian and African cultivated rice were domesticated independently from Asian and African wild rice species, respectively. Here, we constructed a rice graph-based super pan-genome (i.e., a genus-level pan-genome) that integrated gene annotation and position data across 251 genomes spanning various Asian and African wild and cultivated rice species. A super pan-genome can help to discern novel haplotypes for crop potential improvement.^9^ Under the guidance of this super pan-genome, we systematically identified the highly diversified NLRs across rice species and have generated a pan-NLRome for rice. This major resource will inform and motivate plant immunity research for economically relevant cereal grains (which is presently limited to the model eudicot *Arabidopsis*).^24^ The genetic variants revealed in this pan-genome is much more extensive than previous rice pan-genomes. Previous published rice pan-genomes have been constructed either mainly based on short-read DNA sequencing data^4,12^ or based on long-read DNA sequencing data with much smaller sample sizes.^11,13^ The accuracy of InDels and SV identification was greatly improved by the single-molecule sequencing data and high-quality genome sequence enabled us to uncover complex genetic variants.^62^ The discovery of how a set of large DNA fragment deletion events at the *RPAD* locus has influenced plant architecture traits during domestication in both Asian and African rice highlights the significance of this pan-genome as an excellent resource to facilitate future genetic improvement of rice. Although we have sequenced and assembled the largest number of genomes from *Or*, *Og*, and *Ob* accessions using long-read DNA sequencing data, the *Or*, *Og* and *Ob* diversity was not saturated. Considering the high level of genetic diversity present in the wild rice species, more wild rice accessions need to be sequenced in the future.

Given the scope of traits affected by SV, the characterization of SVs is important for understanding phenotypes, adaptation and domestication.^63^ We provided novel examples of how this pan-genome facilitates pinpointing genetic variants that determine agronomic traits, such as grain size or weight, by GWAS and eQTL analysis. Combining different types of genetic variation (such as SNPs, InDels and SVs) will greatly improve the efficiency of association analysis. In addition, the population-scale gene expression profiles are also helpful to find the causal genetic variants that affect gene expression and in turn determine the traits accordingly. We successfully identified a casual genetic variant (i.e., QTN^64^) of TGW (QTL-*qTGW1.2a*) by using both of the pan-SV and gene expression datasets (Fig. 4). In addition, we showed that independent episodes of selection on genes in a genetic network led to the loss of shattering in Asian and African rice. We also showed how selection in African rice for orthologues of submergence-related genes can explain the adaptation of African rice accessions to flood-prone areas. The complete pan-genome information and advances of genome editing tools can reduce barriers in using genetic variants from different cultivated and wild species. The achievement of desirable agronomic traits in cultivated rice and their wild relatives enable realization of breeding by design and the rapid de novo domestication of wild rice species.^65^

To further facilitate exploring this super pan-genome, we developed the Rice Super Pan-genome Information Resource Database (http://www.ricesuperpir.com/) to present and visualize the datasets and provide tools to access these genomic resources. With pan-genome information, it could be more effectively used to identify causal genetic variants (such as SNPs, CNV, PAVs) underlying domestication traits.^63^ However, it is still hard to integrate different types of genetic variations in one graphed pan-genome, especially when it comes to mapping genetic variations identified by short-read sequencing data to the graph. To completely integrate all types of genetic variations in one graphed pan-genome, it will require the development of more efficient tools.

Understanding how the present genomes of different species have been shaped by past evolutionary events will facilitate developing robust strategies for future crop improvement. This rice super pan-genome is a step forward to uncover genetic variants underlying traits, adaptation and domestication in rice, and will provide insights into functional and evolutionary genomics of other crops.

## Materials and methods

### Materials

We collected 251 accessions from 44 countries, including 202 *Os*, 28 *Or*, 11 *Og*, and 10 *Ob* accessions. The *Og*, *Ob*, and *Or* samples were collected because of their geographic diversity. The 202 *Os* samples included 22 elite modern rice varieties, which were collected because of their notable yield, disease resistance, nitrogen use efficiency, and other specific agronomic traits. The rest samples of the *Os* were collected from a MiniCore collection.^14^ The MiniCore was previously collected using a hierarchical sampling strategy. For example, Chinese *Os* MiniCore were collected from 50,526 rice varieties in two steps. The primary core collection consists of 4310 varieties (including 3632 landraces or local varieties, 604 modern pure-line varieties, and 74 parents of trilinear hybrid accessions), which retained approximately 95% of the morphological variation. A MiniCore collection of 189 varieties was further selected from the primary core collection, which retained 70.65% of the simple sequence repeats variation and 76.97% of the phenotypic variation. Detailed information on these accessions is provided in Supplementary information, Table S1a.

### Whole-genome sequencing with nanopore long reads

251 Genomic DNA (gDNA) samples for Nanopore long-read sequencing were extracted from shoots of one-month-old seedlings using a QIAGEN^®^ Genomic DNA extraction kit (Cat #13323, QIAGEN). DNA purity was measured using a NanoDrop™ One UV-Vis spectrophotometer (Thermo Fisher Scientific, USA), which showed that OD_260/280_ ranged from 1.8 to 2.0 and OD_260/230_ was between 2.0 and 2.2. DNA samples were accurately quantified using a Qubit^®^ 3.0 Fluorometer (Invitrogen, USA). Size-selected long DNA fragments were then extracted using the BluePippin system (Sage Science, USA). DNA was then repaired and adapters were attached to the ends using an SQK-LSK109 kit. The concentrations of library fragments were quantified with the Qubit^®^ 3.0 Fluorometer. The DNA library was then loaded into the primed Nanopore PromethION sequencer (Oxford Nanopore Technologies, UK) flow cell. For each accession, the coverage of ONT reads was 98 ± 24× genome coverage. A total of 9.42 Tb of ONT raw reads were obtained (Supplementary information, Table S1a).

### Whole-genome sequencing with Illumina short reads

239 samples were sequenced on the Xten platform (Illumina, San Diego, CA, USA), and 12 gDNA samples for short-read sequencing were from previous publications of our laboratories^66^ (Supplementary information, Table S1a). gDNA samples for short-read sequencing were extracted from leaves of two-week-old seedlings using the CTAB method. Index libraries were constructed with the New England Biolabs (NEB) Next^®^ Ultra™ DNA Library Prep Kit for Illumina (NEB, Ipswich, MA, USA) following the manufacturer’s instructions. Briefly, after quality was assessed, at least 0.2 µg of gDNA from each sample was randomly fragmented by sonication to a size of 350 bp. Then DNA fragments were endpolished, A-tailed, and ligated with the full-length adapter for Illumina sequencing, followed by further PCR amplification. After PCR products were purified by AMPure XP system (Beckman Coulter, Beverly, USA), DNA concentration was measured by Qubit^®^3.0 Flurometer (Invitrogen, USA), libraries were analyzed for size distribution by NGS3K/Caliper and quantified by real-time PCR (3 nM). The clustering of the index-coded samples was performed on a cBot Cluster Generation System using Illumina PE Cluster Kit (Illumina, USA) according to the manufacturer’s instructions. After cluster generation, the DNA libraries were sequenced on Illumina platform and 150 bp paired-end reads were generated. A total of 5.83 Tb raw reads (150 bp paired-end reads) were generated for 239 accessions with a coverage of 65.4 ± 9× (Supplementary information, Table S1a).

### Transcriptome sequencing

Total RNA was extracted from young leaves of one-month-old seedlings (from 249 accessions, except CW01 and CW16) with a TRIzol kit (15596–018). RNA quality was measured with agarose gel electrophoresis, Nanodrop, Qubit 2.0, and Agilent 2100 bioanalyzer. Libraries with a size of 300 bp per insert were constructed using the TruSeq RNA Library Preparation Kit, version 2 (Illumina, USA). RNA was sequenced using the Illumina high-throughput sequencing platform NovaSeq 6000. Finally, a total of 1.85 Tb RNA-seq raw reads were obtained (Supplementary information, Table S1a).

### Hi-C sequencing

gDNA of four accessions (NH229, NH231, NH265, and NH286) were extracted and sequenced for Hi-C analysis. Panicles were respectively collected at the heading stage from NH229 and NH231 accessions. Leaves were respectively collected at the heading stage from NH265 and NH286 accessions. Plant material fixation, nuclei extraction, DNA crosslinking, and restriction enzyme ligation were performed as described previously.^67^ Digested DNA was blunt-ended and incorporated with biotin-14-dCTP (Invitrogen), then ligated with T4 DNA ligase at room temperature for 4 h. After purification, DNA was sheared by sonication with a Covaris S220. Subsequently, end-repaired DNA was separated, purified, and ligated with adapters for library preparation. The final library for Hi-C sequencing was constructed using the *Dpn*II restriction endonuclease, and paired-end sequencing (2 × 250 bp) was conducted on the Illumina NovaSeq 6000 platform. A total of 334.35 Gb raw reads were obtained, NH229, NH231, NH265, and NH286 were 119.6 Gb, 140.1 Gb, 40.0 Gb, and 34.6 Gb, respectively.

### SNP calling

Raw Illumina short reads from gDNA samples were trimmed using Trimmomatic (version 0.36)^68^ with parameters ‘ILLUMINACLIP:2:30:10 MINLEN:75 LEADING:20 TRAILING:20 SLIDINGWINDOW:5:20’. Clean reads were mapped to the Nipponbare^16^ reference genome (MSUv7) using Burrows-Wheeler Aligner (BWA, version 0.7.17–r1188)^69^ with default parameters. SAMtools (mpileup, version 1.8)^70^ was used to generate BCF files. BCFtools (version 1.8)^70^ call was used for SNP calling and filtering (DP < 3 and quality score < 30). SNP calls were further filtered based on the following criteria: (1) integrity ≥ 80% and minor allele frequencies (MAFs) ≥ 0.05; (2) consensus quality ≥ 40; (3) site is diallelic and (4) missing rate < 10%.

### Phylogenetic tree and population structure

The maximum likelihood phylogenetic tree based on SNPs was built using FastTree (version 2.1.11)^71^ with the Jones-Taylor-Thornton CAT model and 20 rate categories. iTOL (version 6.3.1)^72^ was used for a tree visualization (Fig. 1b. The neighbor-joining tree based on SVs (See “SV identification and validation” in Methods), while the PAVs of genes (present and absent non-redundant genes, See “Super pan-genome graph and its annotation” in Methods) were conducted in MEGA (version 7.0.21)^73^ with default parameters.

Maximum likelihood clustering analysis was performed on SNPs of the 251 accessions in ADMIXTURE (version 1.3.0)^74^ using default parameters with K values ranging from 4 to 15.

### De novo genome assembly and evaluation

To get high-quality data, the Nanopore raw reads with quality less than 7 were filtered. The remaining reads were assembled using WTDBG (version 2.5, parameters: -p 0 -k 15 -AS 2 -s 0.05 -L 10000 -l 8192 -e 3).^15^ Contigs were polished once with Nanopore clean reads using wtpoa-cns (version 2.5)^15^ and each polished assembly was further corrected twice with whole genome sequencing 2 × 150-bp pair-end reads using Pilon.^75^ To further improve single base accuracy, NGS short reads were aligned to their assembly with BWA,^69^ and the mutation sites in each accession assembly were identified with FreeBayes (version 1.3.1)^76^ pipeline. Then, variants were filtered with parameters ‘QUAL > 20 & DP > 10 & AO > 10’ and the homozygous sites were replaced.

To remove false duplications from assemblies, Purge Haplotigs (version 1.0.3)^77^ was applied to each assembly with low, middle, and high read depth cutoff tuned artificially. In each 100 kb window, the Nanopore reads depth was plotted against the number of SNPs using a custom R (version 3.1.1) script to detect the redundant sequences (Additional file 4 at https://zenodo.org/record/6602280). The plots are with only one independent cluster, indicating that few, if any, false duplications were present.

To remove various contaminating DNA from archaea, bacteria, viruses, fungi, and other metazoans, the sequences of the protein-coding annotations (see “Gene annotation and expression” in Methods) were aligned to the NCBI Nr database (downloaded on 4 June 2021) with DIAMOND (version 0.9.24)^78^ using parameters ‘-evalue 1e-5’. Contigs in which more than 50% of the sequences of protein-coding annotations aligned to non-viridiplantae organisms were considered contaminants and filtered out. The average GC content (percentage of G and C bases in 10 kb windows) and Nanopore reads depth were used to validate the filtration effect, while the results were displayed using the R package ggplot2 (version 3.3.3)^79^ (Additional file 5 at https://zenodo.org/record/6602280). There was only one independent cluster in these diagrams, suggesting minimal contamination in our assemblies.

Completeness of the assemblies was evaluated through alignment to the Nipponbare^16^ reference genome by MUMmer (version 4.0.0, beta)^80^ with parameters ‘-mum -t 10 -c 90 -l 40’. Syntenic dot plots were visualized with the R package (https://github.com/shingocat/syntenyPlotByR) (Additional file 2 at https://zenodo.org/record/6602280), and the collinearity diagram demonstrates the completeness of the assemblies. Hi-C paired reads from the NH229, NH231, NH265, and NH286 genomes were mapped to their assembled contigs using Juicer (version 1.6).^81^ Contigs were scaffolded using 3D-DNA (version 180922)^82^ to generate draft chromosomes based on alignments with MAPQ score > 20. The draft chromosomes were further manually modified to generate Hi-C heatmaps using Juicerbox (version 1.11.08).^83^

BUSCO (version 9)^17^ evaluation of each assembly showed an average of 96.4% ± 1.6% of the 1,440 single copy Embryophyta genes (Supplementary information, Table S1b), and the BUSCO of Nipponbare^16^ genome was 97.6% using the same way. Continuity was measured by N50 and NG50 contig size. N50 was calculated by Perl script, and NG50 was calculated with QUAST^84^ with Nipponbare genome as reference (Supplementary information, Table S1b). Moreover, the average completeness of each assembly, estimated by 2 × 150 bp pair-end reads of each assembly, was 97.7% ± 0.9% (Supplementary information, Table S1b), which highlights the completeness of the 251 assemblies.

### Gene annotation and expression

A strategy combining ab initio gene prediction, homology-based gene prediction, and RNA-seq was used for gene annotation. For each assembly, repetitive sequences were first masked with RepeatMasker (www.repeatmasker.org, version open-4.0.7) based on RepBase (Edition-20170127, parameter: -species rice). Augustus (version 3.0.3),^85^ SNAP (version 2006–07–28),^86^ and Fgenesh (http://www.softberry.com/) with their default parameters were performed on the repeat-masked genome for annotation with only sequence information. Homologous protein sequences from *Arabidopsis thaliana* (447_Araport11), *Brachypodium distachyon* (314_verison 3.1), *Os* (323_version 7.0), and *Sorghum bicolor* (454_version 3.1.1) were downloaded from Phytozome (https://phytozome-next.jgi.doe.gov/), and all of these sequences were mapped to each assembly with tBLASTN (version 2.9.0+)^87^ with an *E*-value cutoff of 1e−5. Genewise (version 2.4.2, parameter: -gff -quiet -silent -sum)^88^ was used to refine the alignment. Raw RNA-seq reads from leaf tissue of 249 accessions (except CW01 and CW16) were trimmed by Trimmomatic^68^ with parameters ‘ILLUMINACLIP:TruSeq3-PE.fa:2:30:10 LEADING:3 TRAILING:3 SLIDINGWINDOW:4:15 MINLEN:36’. RNA-seq reads were aligned to the corresponding genomes with HISAT2 (version 2.1.0, default parameter)^89^ and assembled into transcripts with StringTie2 (version 2.1.4, default parameter).^90^ Open reading frames (ORFs) were predicted using TransDecoder (version 5.5.0).^91^ For CW01 and CW16, which lacked corresponding RNA-seq data, we used RNA-seq data from CW02 and CW15 as transcriptomic evidence. All results were integrated into consensus gene models using EvidenceModeler (version 1.1.1).^92^

Representative proteins (translated from the longest isoforms) of genes from 251 accessions, together with Nipponbare,^16^ R498,^93^ *Amborella trichopoda* (291_V1.0), and *Brachypodium distachyon* (314_V3.1) were clustered into orthogroups (OGs) based on sequence similarity using OrthoFinder (version 2.2.6).^94^ Nipponbare and R498 are typically used as the reference genomes for *Osj* and *Osi* in rice functional research. Both *A. trichopoda*^95^ and *B. distachyon* were used as outgroups in clustering genes by OrthoFinder.^94^ *A. trichopoda* did not experience whole-genome duplication and *B. distachyon* was a model plant for *Poaceae Barnhart* plant. Proteins of *A. trichopoda* and *B. distachyon* were downloaded from Phytozome.

To verify the PAVs of OGs (the PAVs across 251 accessions to the genomes absent of the OGs), we recovered the genes in OGs across the 251 rice accessions and mapped to each genome following the methods of a recent study:^96^ (1) A preliminary gene sets with complete gene structure was obtained. We aligned the coding sequence of genes in OGs with PAVs across 251 accessions to the genomes absent of the OGs using GMAP (version 2017–11–15).^97^ Alignments were filtered to have ≥ 90% coverage and ≥ 90% identity (parameters ‘-min-trimmed-coverage=0.9 -min-identity=0.9’, version 2017–12–15); (2) The preliminary gene sets were filtered to remove pseudogenes identified using the PseudoPipe^98^ program, which was a homology-based computational pipeline, includes two steps: (i) using protein sequences to find pseudogenes in repeat-masked intergenic regions by tBLASTN;^87^ and (ii) realignment of candidates to corresponding parent(s) by FASTA^99^ to validate and classify pseudogenes. The preliminary genes with coverage > 0.7, identity > 0.4 and *E*-value < 1e−10 were considered as pseudogenes and were filtered out. Each genome recovered 1288 ± 89 (3.68% ± 0.25%) confidence genes; (3) The authentic gene set was then evaluated with BLASTP^87^ and transcriptomic data. The protein sequences of the authentic genes for the OGs were aligned with the present protein sequences of corresponding OGs using BLASTP,^87^ and the sequence with coverage > 0.9 and identity > 0.9 and *E*-value < 1e−10 were considered high-confidence genes and were kept. Then the high-confidence genes with RNA-seq coverage > 0.5 were considered final target genes, and each genome recovered 293 ± 45 (0.84% ± 0.13%) genes with high confidence and transcriptomic evidence; (4) This gene set was merged with the original gene annotation result by EvidenceModeler^92^ to produce the final gene annotation for each assembly (Supplementary information, Table S1c). We also updated the PAV matrix based on the updated annotations. After validation, OGs were called non-redundant genes in the following analysis.

To assess the completeness of the genome annotations, the number of the identical genes in 9311 (NH231), IR64 (NH236), and N22 (NH241) from Qin et al.^11^ and our study was compared. More than 99% of the genes are identical. The detailed processes are as follows. First, we compared the sequences assembled in this study and reported by Qin et al.^11^ using MUMmer^80^ (parameters: NUCmer -c 90 -l 40). The alignment blocks were filtered with one-to-one alignment mode (-1). The filtered alignment results were then used to calculate the unmatched sequence by BEDTools (version 2.29.1)^100^ complement command, and based on the annotation of each genome, the command BEDTools intersect^100^ was applied to calculate the number of genes that were wrapped in the sequences on the alignment.

Gene expression levels (fragments per kb of exon per million mapped fragments (FPKM)) were calculated from the HISAT2^89^ alignment results using Cufflinks^101^ with default parameters mapping to each corresponding assembly.

### Repeat sequence annotation

Repeat sequences of the 251 rice assemblies were annotated by Extensive de novo TE Annotator (EDTA, version 1.9.6)^18^ composed of eight published programs. More specifically, LTR_Finder (version 1.07),^102^ LTRharvest (version 1.5.10)^103^ and LTR_retriever (version 2.9.0)^104^ were incorporated in this package to identify LTR retrotransposons. Generic Repeat Finder (version 1.0)^105^ and TIR-Learner (version 1.23)^106^ were included to identify TIR transposons; HelitronScanner (version 1.0)^107^ identifies Helitron transposons. RepeatModeler was used to identify TEs (such as SINEs and LINEs) missed by the other structure-based programs, and RepeatMasker was used to annotate fragmented TEs based on homology to structurally annotated TEs. The curated TE library (rice 6.9.5.liban) from the EDTA^18^ package was used to annotate whole-genome TEs in the 251 rice assemblies with parameters ‘-overwrite 1 -sensitive 1 -anno 1’ (Supplementary information, Table S1d).

### Super pan-genome graph and its annotation

Minigraph (version 0.15-r426)^108^ with option ‘-xggs’ was used to integrate the 251 genome assemblies and Nipponbare^16^ into a multi-assembly graph. We separately aligned each assembly back to the pan-genome graph to obtain allele information for each bubble (a locus with variants in the pan-genome graph), including the path, strand, contig start, and contig end through the bubble in each assembly (alignment parameters: minigraph -xasm -call). The approximate locations of bubbles uncalled by minigraph^108^ were inferred from the nearest two collineated bubbles. Based on the allele information from each assembly for each bubble, we obtained the collineation location of all assemblies.

To integrate gene annotation with the pan-genome graph, the bubbles, overlapped with the genes on a corresponding assembly were extracted using the command BEDTools intersect.^100^ The pan-genome location and allele information (contig name, contig start and contig end through the bubble) of these bubbles were then extracted. Bubbles that were not collineated with other bubbles on both the corresponding assembly or the pan-genome graph were filtered out. The location of each gene on the pan-genome graph was inferred based on the location of overlapping bubbles. Results are shown in the database (http://www.ricesuperpir.com/).

To select the non-redundant novel sequences of each sub-population, the redundant sequences of sequence segments in no-reference paths of each sub-population were removed using a cutoff of 90% identity and 90% coverage by minimap2 (version 2.17-r974-dirty)^33^ with the command ‘minimap2 -ct 4 -dual=no -D’. Non-redundant sequences were further assessed with minimap2^33^ using Nipponbare^16^ as the reference (parameters: -ct 4 -dual=no -D) at a cutoff of 90% identity and 90% coverage. The remaining sequences formed the non-redundant novel (non-reference) sequences of each sub-population. The annotations of each non-redundant novel sequence were extracted from the corresponding assembly annotation.

Average numbers of non-redundant genes that were different between any two accessions were evaluated by calculating the average of numbers of gene with PAVs between two randomly selected accessions. The average ratio of numbers of gene with PAVs between two randomly selected accessions and the average gene numbers between them were also calculated.

The presence and absence of several known genes (*Hd1*,^109^ *OsSh1*,^20^ *Pit*,^110^ and *OsLCT1*^21,111^) were validated by PCR. Primers are shown in Supplementary information, Table S6a.

We defined the non-redundant genes present in ≥ 95% of 251 accessions as core non-redundant genes and those present in < 95% as dispensable non-redundant genes. Distribution of *Oryza* genes were inferred by the present pattern of genes in different accessions. All non-redundant genes from our pan-genome were grouped into 4 taxonomic levels (I: Genes present in both *Or* and *Ob*, II: Genes present in both *Or* and *Os* except genes of level I, III: Genes present in both *Og* and *Ob* except genes of level I, IV: Genes present in both *Osi and Osj* except genes of level I and level II). However, some genes could not be clearly determined, e.g., genes only present in *Osi* and *Og* (likely due to admixture).

The total number of non-redundant genes present in a population was estimated based on simulations. We evaluated how the total number of non-redundant genes changed when new accessions were included (Supplementary information, Fig. S2b, c). To achieve this, we randomized the order of rice accessions 500 times. Each time, we counted the observed number of non-redundant genes when a new accession was added (according to the randomized order). The results of 500 times simulation were used for the plot using MATLAB (R2016a). We utilized a different set of genes in this analysis: (1) all genes, non-private genes (non-private genes are defined as non-redundant genes present in at least two accessions) in 230 Asian accessions, and (2) all genes and non-private genes (non-private genes are defined as non-redundant genes present in at least two accessions) in 21 African accessions. This analysis estimates the proportion of genes that could be captured by accessions used in this study, e.g., the total number of Asian rice genes approached a plateau.

### Variation graph and its application

To facilitate exploration of the super pan-genome in an efficient way, we used 159,491 DELs and INSs from the pan-SV dataset (against Nipponbare^16^ reference genome; See “SV identification and validation” of Methods for detailed information). The high-quality Nipponbare^16^ and these INSs/DELs were used to construct the graph-based genome by vg toolkit,^112^ The short reads of 605 *Os* accessions (PRJCA000322)^43^ were filtered using Trimmomatic^68^ with the parameters ‘MINLEN:50 LEADING:20 TRAILING:20’. The filtered short reads of each accession were mapped to the variation graph to call variants using vg toolkit^112^ with parameters ‘map; pack -Q 20; call’.

### Whole-genome alignment

Whole-genome alignment of all assemblies was performed using Cactus (version 1.3.0).^23^ To build the guide tree for Cactus alignment, whole-genome SNPs of 256 rice accessions (including 251 sequenced accessions in the study, NIP,^16^ R498,^93^ one *O. glumaepatula* accession, one *O. meridionalis* accession, and one *O. punctata* accession) were used to generate a putative phylogenetic tree following a standard protocol in FastTree.^71^ Information about the genomes of R498, *O. glumaepatula*, *O. meridionalis* and *O. punctata* accessions are in Supplementary information, Table S6b. To provide outgroup information for sub-problems near the root, the genomes of *O. glumaepatula*, *O. meridionalis*, and *O. punctata* were used in phylogenetic tree construction but were removed from the final alignment. To ensure that a high-quality assembly was always available as an out-group, NIP^16^ and R498^93^ were marked as preferred outgroups. Repetitive sequences were softmasked by RepeatMasker based on RepBase (Edition-20170127). We then ran Cactus^23^ for each sub-tree with 20–23 accessions on a single node and obtained the final alignment in a step-by-step manner. Cactus^23^ alignment-related results, pan-genome variations and genome annotations are shown on http://www.ricesuperpir.com/.

### Database construction

The Rice Super Pan-genome Information Resource Database (RiceSuperPIRdb) was constructed to display and manage the rice pan-genome. The basic architecture of RiceSuperPIRdb consists of an Apache HTTP web server (http://www.apache.org/), MySQL (http://www.mysql.com/) data management system, SpringCloud framework (https://flask.palletsprojects.com/en/1.1.x/), and a popular front-end component library, Bootstrap (http://getbootstrap.com/). The rice genome assemblies, annotations, pan-genome variations, TE annotations, and Cactus^23^ alignment-related results were displayed in JBrowse^113^ and were obtained by loading our assembly results into our genome browser by copying the hub link (http://www.ricesuperpir.com/) into the PAN BROWSER page. A BLAST tool was also constructed using SequenceServer^114^ and BLAST.^87^ There are no restrictions on use. Other related data to the 251 rice accessions used in this study can also be viewed and accessed at http://www.ricesuperpir.com/.

### The analysis of pan-NLRome

Genes containing the NB-ARC domain were identified by InterProScan^115^ with *E*-value < 1e−4. The results of NLR-Parser (version 1.0)^116^ and InterProScan^115^ were cross-verified and supplemented to determine the LRR domain. Genes containing the NB-ARC domain and any one of the three motifs in 9, 11, and 19,^116^ which are always common on rice were identified as NLRs.^117^

Then we verified the results of NLRs with two approaches to ensure the accuracy of subsequent analysis. First, we applied the NLR identification method to the Nipponbare reference genome to estimate the feasibility of NLR capturing. All currently known and cloned NLRs from Nipponbare were obtained, proving that the NLR identification method is feasible (Supplementary information, Table S2d). We also compared the number of NLRs in each accession with the published the number of NLRs found in the Nipponbare (including 499 NLRs) and the Tetep genomes^117^ (including 455 NLRs). We found that the number of NLRs in these genomes were similar, suggesting that the NLR annotation results are reliable (Supplementary information, Table S2a).

The identification of integrated domains was based on the other domains obtained from InterProScan.^115^ We counted the frequency of integrated NLR domain within each sub-population (FDR < 0.01; Wilcoxon tests, Supplementary information, Table S2b), and focused on domains with a significant difference in frequency distribution (Supplementary information, Fig. S3a). Then, in order to determine how NLRs were regulated, the expression levels of NLRs under standard growth conditions were carried out.

To determine the characteristic information of NLRs in the pan-NLRome, non-redundant NLRs and their evolutionary relationship were obtained from the non-redundant genes of the pan-genome. In each sub-population, the core non-redundant NLRs were defined as those present more than 95% of all the genomes, whereas the rest of genes were defined as dispensable.

Most of the identified functional NLRs did not exist alone, resulting in the introduction of NLR pairs and clusters.^7,117^ If there were fewer than two non-NLR genes between any two NLRs, these two genes were defined as “approaching”. Three or more approaching NLRs formed a cluster, whereas two approaching NLRs were considered as a pair. The remaining NLRs (without approaching NLRs) were labeled singleton NLRs.^117^

Combined with the classification results of the non-redundant genes, the internal homogeneity and heterogeneity of NLR pairs and clusters were further elucidated. If all genes in each pair or cluster belonged to the same non-redundant genes, this pair or cluster was considered as homogeneous; if there were two or more non-redundant genes, the pair or cluster was regarded as heterogeneous.^7^ The orientation of NLRs within a pair was also analyzed. Two paired NLRs in the same direction were classified as T-H. The H-H orientation assumes the two promoters reside near each other, whereas the T-T orientation assumes they are on the outer ends of the region.^117^

Plots were generated with R (version 3.6.0) packages with ggplot2,^79^ ggpubr (version 0.4.0),^118^ and ggpmisc (version 0.4.0).^119^

### Collinearity of NLR singletons, pairs and clusters

To get the genomic collinearity of NLR singletons, pairs, and clusters in all assemblies, the super pan-genome graph by minigraph^108^ was employed, which contained the collinearity of all assemblies. We used the Nipponbare genome^16^ as a reference (i.e., the pan-genome location) to show the collinearity of all assemblies. First, bubbles overlapped with the NLR singletons, pairs, and clusters in a corresponding assembly were extracted using the command BEDTools intersect.^100^ Then the pan-genome location and allele information (contig name, contig start, and contig end through the bubble) of these bubbles were extracted. Bubbles that were not collineated with other bubbles on both the corresponding assembly and the pan-genome graph were filtered out. The location of NLR singletons, pairs, and clusters on the pan-genome graph was inferred based on the location of overlapping bubbles. Finally, once the pan-genome locations of NLR singletons, pairs, and clusters of different assemblies were overlapped (identified using the command ‘BEDTools cluster’), they were labeled on the same locus in the pan-genome graph. If two NLR singletons fell in the same bubble, only one was kept for comparison across all accessions to identify the locus; the remaining singleton was put on a new locus 1 bp away from the original locus. If the locus only contained a singleton NLR, a single pair, or a single cluster gene, it was excluded from further analysis.

The collinearity accuracy of NLR loci shown in the main text was confirmed by aligning the NLR loci sequence of each assembly with each other using MUMmer^80^ (parameters: NUCmer -c 90 -l 40). The alignment blocks were filtered using a delta-filter with one-to-one alignment mode (-1). The collinearity of these NLR loci on the *O. longistaminata*^120^ genome was obtained by aligning the NLR loci sequence with the *O. longistaminata*^120^ genome using MUMmer.^80^ The R packages RIdeogram (version 0.2.2)^121^ and genoPlotR (version 0.8.11)^122^ were used for collinear plot.

### SV identification and validation

To call SVs (DELs, INSs, INVs, TRAs, and DUPs), high-quality Nanopore reads of the 251 accessions were aligned to the Nipponbare^16^ genome using minimap2^33^ and NGMLR (version 0.2.7).^34^ SVs were called using Sniffles (version 1.0.11, parameters: -l 50 –genotype).^34^ SVs were removed if they (1) were larger than 1 Mb or smaller than 50 bp; (2) fell in the gap region of the reference genome; (3) showed a “0/0” genotype; (4) were labeled “IMPRECISE” by sniffles or (5) had a read depth < 30. SURVIVOR (version 1.0.7)^123^ (parameters: 1000 2 1–1–1 50) was used to merge SVs called by both NGMLR^34^ and minimap2^33^ in each accession (Supplementary information, Fig. S4a and Table S3b).

To quantitatively estimate the accuracy of SV calling, we manually examined 500 randomly selected SVs by visualizing the corresponding long-read alignment through an Integrative Genomics Viewer (IGV, version 2.9.2)^124^ Browser. The SV calling accuracy was estimated to be 95.8% (Supplementary information, Table S3c). Large SVs were manually validated using a chromatin interaction heatmap at 5 kb resolution based on Hi-C sequencing data of four accessions by Juicer^81^ (Supplementary information, Table S3c). For instance, the Hi-C reads from NH229, NH231, NH265, and NH286 were mapped to the Nipponbare^16^ reference genome. The reads with a MAPQ quality score < 30 were discarded, and the Hi-C contact heatmaps were visualized using Juicerbox.^83^ In addition, SVs within genes including *RFT1*,^125^ *HGW*,^37^ *OsNaPRT1*^38^ were validated by PCR (Supplementary information, Fig. S5n and Table S6a). Primers are shown in Supplementary information, Table S6a.

SVs relative to Nipponbare genome were used for analyses of SV hotspots, GWAS, and ADMIXTURE.

### SV characteristic analysis

The total number of SVs present in a population was estimated based on simulations. We evaluated how the total number of SVs changed when a new accession was included (Supplementary information, Fig. S4f, g). To achieve this, we randomized the order of rice accessions 500 times. Each time, we counted the observed number of SVs when a new accession was added (according to the randomized order). The results of 500 times simulation were used for the plot using MATLAB (R2016a). We utilized a different set of SVs in this analysis: (1) all SVs, non-private SVs (non-private SVs are defined as SVs present in at least two accessions) in 230 Asian accessions, and (2) all SVs and non-private SVs in 21 African accessions. This analysis estimates the proportion of SVs that could be captured by accessions used in this study, e.g., the total number of Asian rice SVs approached a plateau.

### SV genomic feature annotation

Throughout the manuscript, we describe various relationships between SVs and other genomic features such as genes and intergenic regions. Generally, we annotated the pan-SV sets with genomic features using Vcfanno (version 0.3.2)^126^ with default parameters. Vcfanno^126^ annotated SVs by finding their intersection (overlap) with genomic feature intervals. Accordingly, some annotations reported in the Nipponbare genome^16^ could be directly interpreted from Vcfanno,^126^ including gene and intergenic region (Supplementary information, Fig. S4k). To detect genes contained by SVs, we first checked if the gene start and end positions intersected a given SV. If that SV intersected both the start and end of a gene, it contains that gene.^127^

### Identification of SV hotspot regions

Pan-SV sets of the *Osi*, *Osj*, *Or*, *Og*, and *Ob* sub-populations were respectively merged by SURVIVOR^123^ (parameters: 1000 1 1–1–1 50). We calculated the distribution of SV breakpoints for each 200 kb window (with a 100 kb step size) along each chromosome for each sub-population.^11^ Then, all 200 kb windows were ranked in descending order according to the numbers of SVs within the window, the top 10% of all windows with the highest frequency of SV breakpoints were defined as SV hotspots. All of the continuous hotspot windows were merged as hotspot regions^11^ (Supplementary information, Fig. S4j).

To estimate the genomic background of SV content, we simulated SVs with their sizes matched to the real variants 100 times in five sub-populations. In each simulation, 76,898 SVs, 45,736 SVs, 122,444 SVs, 34,210 SVs and 42,575 SVs respectively from *Osi*, *Osj*, *Or*, *Og*, and *Ob* sub-populations pan-SV set were randomly generated against the Nipponbare^16^ genome. The SV number difference of each window between variants and simulated variants was calculated with the Wilcoxon test, the *P-*values of *Osi*, *Osj*, *Or*, *Og*, and *Ob* sub-populations were 1.7e−180, 8.80e−219, 2.1e−40, 2e−43 and 1.7e−31, respectively.

### GWAS

GWAS was conducted using two different datasets. On one hand, the SNPs, insertions, and deletions from our dataset were filtered by VCFtools (version 0.1.13)^128^ with missing rate < 0.1, allele frequency ≥ 0.05, and no multiple alleles. Phenotypes in our dataset comprised grain length and 1000-grain weight surveyed at the harvest stage in Lingshui of Hainan Province, China at 2020. Principal component analysis (PCA) was conducted by plink2 (version 2.00a3LM)^129^ (parameters: -allow-extra-chr -pca 10). The first five principal components and standardized matrix of kinship (GEMMA,^130^ version 0.98.1 -gk 2) were used as covariates. GWAS was performed using a mixed linear model in genome-wide efficient mixed model association software (GEMMA, parameters: -lmm 4 -k) with the *O. sativa* population. The threshold for GWAS was calculated using a Genetic Type I error calculator (GEC)^131^ at α = 0.05 level.

On the other hand, GWAS was also conducted using SVs inferred by vg toolkit^112^ using the previously reported dataset^43^ (See “Variation graph and its application” in Methods). SNPs and phenotypes (grain yield) were acquired from the same source (Supplementary information, Table S4). Both markers were also filtered by VCFtools^128^ with a missing rate < 0.1, allele frequency ≥ 0.05, and no multiple alleles. GWAS was conducted in the same way as the current dataset described here.

### eQTL analysis

Clean RNA-seq reads were mapped to the Nipponbare genome^16^ with TopHat2 (version 2.0.12).^132^ Based on the alignments, raw read counts were derived for each gene and normalized to FPKM^133^ using Cufflinks^101^ with default parameters. For eQTL analysis, expression data from 202 *Os* accessions were used. Genes with a mean FPKM value larger than 0.1 were used in the downstream analysis and 23,736 genes met this condition. To obtain a normal distribution of expression values for each gene, FPKM values of each gene were further normalized using the quantile-quantile normalization (qqnorm) function in R (version 3.1.2). The top 20 hidden and confounding factors in the expression data, the normal quantile transformed expression values were inferred using the probabilistic estimation of expression residuals (PEER) method.^134^ There were 39,027 SVs with allele frequency ≥ 0.05 and max-missing ≤ 0.1 filtered by VCFtools^128^ for downstream analysis. PCA was conducted to infer population structure (plink2 -allow-extra-chr -pca 10). Both the first 20 factors in PEER results and the first five principal components in PCA analysis were used as covariates. The linear regression model of the MatrixEQTL package (version 2.2)^135^ was used to detect associations between SV-gene pairs. *P*-values corrected by the Benjamini-Hochberg method at α = 0.05, and *P* = 1.29e−5 were used as the genome-wide error threshold (Calcuated by P. adjust package (version 3.1.2)^136^ in R).

Linkage disequilibrium (LD) decay was measured using PopLDdecay (version 3.41, parameters: -MaxDist 500 -MAF 0.05 -Het 0.88 -Miss 0.999).^137^ The stable *r*^2^ value (0.11 for deletions and insertions) was considered as the background level of LD. Candidate eQTL blocks were selected as described previously.^138^ In brief, for multiple eQTLs associated with the same gene, if *r*^2^ between two eQTLs was larger than the stable *r*^2^, the site with a smaller *P*-value would be retained. If an SV resides within the corresponding gene or is less than 2 kb from the transcriptional start site or the end of a gene, it was classified as an eQTL.

### Domestication and differentiation

Both the relative divergence measure *F*_ST_ and the absolute genomic divergence measure *D*_*xy*_ were estimated to identify domestication and differentiation regions. *D*_*xy*_ and Per-site Weir-and-Cockerham *F*_ST_ were calculated using PBScan (version 1.0)^139^ and VCFtools,^128^ each with 20 kb sliding windows. *D*_*xy*_ and *F*_ST_ values were ranked, and windows with the top 5% of values were selected as highly divergent regions.

### Phylogenetic analysis of *RPAD*

The cDNA sequences of *ObZNF1−ObZNF10* (from NH278) and *OrZNF1−OrZNF8* (from DXCWR, https://www.ncbi.nlm.nih.gov/nuccore/MF503970) (Supplementary information, Fig. S7f) were subjected to multiple sequences alignment using CLUSTALW (version 2.1).^140^ Based on the alignment results, FastTree^71^ was used to conduct phylogenetic analysis (Supplementary information, Fig. S7f) and iTOL^72^ was used to visualize.

To analyze the structure and evolution of proteins ObZNF1, OrZNF1, ObZNF10, and OrZNF8, the protein sequences from NH278 and DXCWR^60^ (https://www.ncbi.nlm.nih.gov/nuccore/MF503970) were used for alignment using CLUSTALW (version 2.1).^140^

### Functional genomic analyses

To validate the function of the candidate genes, QTL-*spd6*,^42^ and QTL-*qTGW1.2a*,^39^ NIL-*qTGW1.2a*^NIP^ and NIL-*spd6*^*Or*^ were developed. An NIL that carries the *qTGW1.2a* Hap.1 allele from NIP was constructed by backcrossing with the *indica* variety 9311. To develop NIL-*spd6*^*Or*^, Zhonghua 11 (ZH11) was used as the recurrent parent and CSSL58 (in *Os*. cv Teqing background with the *spd6*^*Or*^ introgression) was used as donor parent, then the advanced backcross line showing a similar CSSL58^42^ genotype with short grain was regarded as NIL-*spd6*^*Or*^ (in ZH11 background).

To validate the expression levels of *LOC_Os01g57250* in 9311, NIL-*qTGW1.2a*^NIP^, and NIP, RNA isolation and qPCR were conducted. Total RNA was extracted from seedlings of the lines using TRIzol reagent (Invitrogen), then the RNA was reversely transcribed to cDNA using RT SuperMix for qPCR (Vazyme) following the manufacturer’s instructions. qPCR was performed using SYBR^®^ qPCR Master Mix (Vazyme). Three independent RNA samples for each line were used as biological replicates. Primers for qPCR are listed in Supplementary information, Table S6a.

To generate the pCAMBIA1301-35SN-*spd6* over-expression construct, the cDNA fragment of *spd6* (*LOC_Os06g04820*)^42^ was amplified from NIP and cloned into pCAMBIA1301-35SN. Then, the over-expression vector of *spd6* (*LOC_Os06g04820*) was transferred into a NIL-*spd6*^*Or*^ line, which was developed by backcrossing with the *japonica* variety ZH11. The genomic fragments of nine zinc finger genes (*ObZNF1*–*ObZNF9*) were acquired from NH278 or NH283 by PCR amplification (Supplementary information, Table S6a), and inserted into the binary vector pCAMBIA1300 with a homologous recombination method to generate complementary vectors (*ObZNF1-*CP to *ObZNF9*-CP). We introduced these vector plasmids into *Agrobacterium tumefaciens* strain EHA105 using a freeze-thaw method. Complementary vectors of nine zinc finger genes were transferred into GIL28. Primers for vector construction are provided in Supplementary information, Table S6a.

A phenotypic investigation of plant architecture of transgenic lines of *ObZNF1*–*ObZNF9* was performed during the tillering stage using 10 individual plants from positive transgenic lines of the nine zinc finger genes. Besides, agronomic traits including grain length and 1,000-grain weight for transgenic lines, NIL lines of *spd6*,^42^ and NIL lines of *qTGW1.2a*^39^ were surveyed at the rice harvest stage.

Nanopore sequencing and Sanger sequencing were used to validate the genotype of NIL-*spd6*^*Or* 42^. The primers used for PCR were listed in Supplementary information, Table S6a. The genomic DNA sample for Nanopore and NGS sequencing was extracted from leaves of a three-month-old NIL-*spd6*^*Or*^ line. The subsequent procedures were conducted as described in “Whole-genome sequencing with nanopore long reads”, “Whole-genome sequencing with illumina short reads” and “De novo genome assembly and evaluation” in Methods.

### Haplotype analysis

We analyzed the haplotype pattern of several known submergence tolerance-related genes in five sub-populations. The sequences including *SNORKEL1* (AB510478.1),^49^ *SNORKEL2* (AB510479.1)^49^ and *Sub1A* (DQ011598.1)^52^ were downloaded from NCBI (https://www.ncbi.nlm.nih.gov/nuccore/). The sequences including *Sub1B* (*LOC_Os09g11480*),^52^ *Sub1C* (*LOC_Os09g11460*),^52^ *DEC1* (*LOC_Os12g42250*),^51^ *ACE1* (*LOC_Os03g22510*)^51^ and *ACE1-LIKE1* (*LOC_Os07g47450*)^51^ were downloaded from the MSU Rice Genome Annotation Project Database (http://rice.uga.edu/). Then, these gene sequences were captured in each accession of five sub-populations. Since the absence of *SNORKEL1*, *SNORKEL2*, and *Sub1A* in Nipponbare genome sequences,^16^ the *O. rufipogon* (IRGC106162)^141^ and NH231 with scaffold sequences were used as reference genomes. The sequences of *SNORKEL1* and *SNORKEL2* were aligned to *O. rufipogon* (IRGC106162) and the sequences of *Sub1A* were aligned to the assemblies of NH231 with BLASTN^87^ to get their flanking sequences (SAMtools^70^ faidx). *Sub1B*, *Sub1C*, *DEC1*, *ACE1*, and *ACE1*-*LIKE1* and the flanking sequences (from upstream 5 kb to downstream 5 kb) were intercepted from Nipponbare genome.^16^ The submergence tolerance-related genes with their flanking sequences were aligned to the genome sequences of each accession (BLASTN, max_target_seqs 3) to get genomic sequences of the genes in every accession (SAMtools^70^ faidx). And then, the complementary DNA sequences of the genes were aligned to their genome sequences with flanking sequences of each accession (BLASTN, identities > 90, *E*-value = 1e−10). When the ratio of alignment length to the gene length was > 0.9, the gene was identified to be present in the accession. For the genes including *SNORKEL1*, *SNORKEL2*, *Su1A*, *Sub1B*, *Sub1C*, *ACE1*, and *ACE1-LIKE1* that were present in the accessions, the haplotypes were distinguished based on the sense mutation in the exon region. For gene *DEC1*, the haplotypes were distinguished dependent on a 7 kb DEL in 9 kb upstream of *DEC1* and a 54 bp INS in exon region.

The collinearity accuracy of known submergence tolerance related genes (*SNORKEL1*/*2*),^49^ *Sub1A*/*B*/*C*^52^ shown in the main text was confirmed by aligning the gene sequences of each assembly with each other using MUMmer (version 4.0.0, parameters: NUCmer -c 90 -l 40).^80^ The alignment blocks were filtered using a delta-filter with one-to-one alignment mode (-1). The collinearity location of these known genes on the *O. longistaminata*^120^ genome was obtained by aligning the *SNORKEL1*/*2*, *Sub1A*/*B*/*C* sequences with the *O. longistaminata*^120^ genome using MUMmer. The R packages genoPlotR (version 0.8.11)^122^ were used for collinear plot.

### Quantification and statistical analysis

All details of the statistics applied are provided alongside in the figure and corresponding legends and methods. All statistics were carried out in R using Student's *t*-test or Wilcoxon rank sum and signed rank tests where appropriate (unless otherwise indicated). For normally distributed variables, one-way analysis of variance (ANOVA) with Tukey’s test was performed using multcomp (version 1.4–16)^142^ package in R. For non-normally distributed variables, Kruskal-Wallis test with Bonferroni’s multiple comparison post hoc test was performed using agricolae (version 1.3–5)^143^ package in R. Normal distribution was tested using the function by f.shapiro with RVAideMemoire package (version 0.9–80)^144^ in the R.

## Supplementary information

Fig. S1

Fig. S2

Fig. S3

Fig. S4

Fig. S5

Fig. S6

Fig. S7

Table S1

Table S2

Table S3

Table S4

Table S5

Table S6

## Data Availability

All data released with this study can be freely used. We are organizing phylogenomic analyses and other analyses using the whole-genome alignment, and we encourage researchers to contact us for collaboration. Genome sequencing data of 251 accessions in this study have been deposited in the NCBI Sequence Read Archive (https://www.ncbi.nlm.nih.gov/sra) under BioProjects PRJNA656318 and PRJNA692836. Genome sequencing data of NIL-*spd6*^*Or*^ in this study have been deposited in the NCBI Sequence Read Archive (https://www.ncbi.nlm.nih.gov/sra) under BioProjects PRJNA764059 and PRJNA764040. All rice assemblies and annotations in this study have been deposited at the Genome Warehouse (GWH) (https://bigd.big.ac.cn/gwh/) under PRJCA004295. The RiceSuperPIRdb browser (http://www.ricesuperpir.com/) integrated the super pan-genome graph, annotation of 251 rice assemblies, alignment of whole-genome annotation of 251 rice accessions, SVs information of 251 rice accessions and variation graph. Four Hi-C datasets have been deposited in the NCBI Sequence Read Archive under PRJNA693366. The RNA sequencing data used in this study have been uploaded to NCBI SRA under accession PRJNA692672. The additional files for the diagrams are available in https://zenodo.org/record/6602280, including population structure, GC content and Nanopore reads depth, highly divergent regions, SNP number and Nanopore reads depth, and collinearity of assembled genome against Nipponbare reference genome. RepBase (Edition-20170127) was downloaded online (https://www.girinst.org/). InterProScan5 database was downloaded online (https://interproscan-docs.readthedocs.io). Homologous proteins were downloaded from Phytozome (https://phytozome.jgi.doe.gov/pz/portal.html).

## Code availability

All data were analyzed with standard programs and packages, as detailed above. Code is available on request.
