# Supplementary material for: A super pan-genomic landscape of rice
Source: Cell Res. 2022 Jul 12;32(10):878–96. doi: 10.1038/s41422-022-00685-z (PMC9525306; doi:10.1038/s41422-022-00685-z)
Supplement: Supplementary file 1 — Fig. S1 [file 41422_2022_685_MOESM1_ESM.pdf]

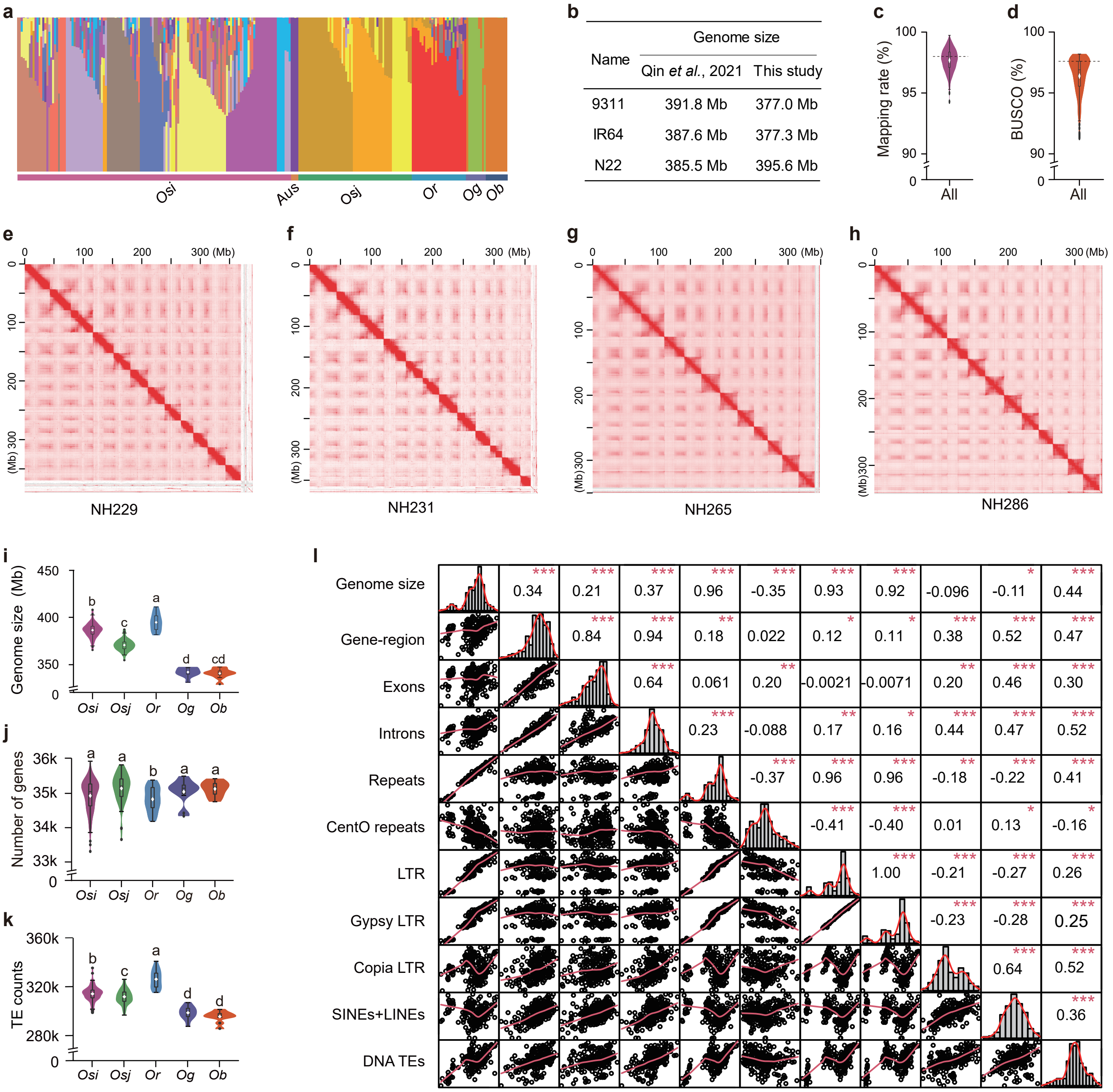

Fig. S1. Population structure, assembly assessment and annotation of 251 accessions. a Population structure of 251 rice accessions inferred by ADMIXTURE. The length of each segment in each vertical bar represents the proportion contributed by ancestral populations (K=15). **b** Genome size of 9311 (NH231), IR64 (NH236), and N22 (NH241) previously reported and assembled in this study. **c** Mapping rate of Illumina Next-generation sequencing (NGS) reads. The dashed line indicates rate of Nipponbare NGS reads mapped to its genome. **d** BUSCO evaluation of assemblies across the 251 accessions. The dashed line indicates BUSCO result of Nipponbare reference genome. **e-h** Hi-C contact maps of NH229 (**e**), NH231 **(f**), NH265 (**g**) and NH286 (**h**) (500 kb bins). **i** Comparison of genome size across different sub-populations. **j, k** The number of protein-coding genes (**j**) and TEs (**k**) across different sub-populations. TEs and LTR refer to transposable elements and long terminal repeat, respectively. **l** Spearman correlation coefficients and *P*-values for comparisons between genome size and sizes of other features: gene-region, exons, introns, repeats, CentO repeats, LTR, Gypsy LTR, Copia LTR, SINEs+LINEs, and DNA TEs in 251 accessions, respectively. * $P < 0.05$, ** $P < 0.01$, and *** $P < 0.001$. *Osi*, *Aus*,*Osj*, *Or*, *Og*, *Ob* refer to *O. sativa indica*, *O. sativa aus*, *O. sativa japonica*, *O. rufipogon*, *O. glaberrima*, *O. barthii*, respectively.
