# Supplementary material for: A super pan-genomic landscape of rice
Source: Cell Res. 2022 Jul 12;32(10):878–96. doi: 10.1038/s41422-022-00685-z (PMC9525306; doi:10.1038/s41422-022-00685-z)
Supplement: Supplementary file 2 — Fig. S2 [file 41422_2022_685_MOESM2_ESM.pdf]

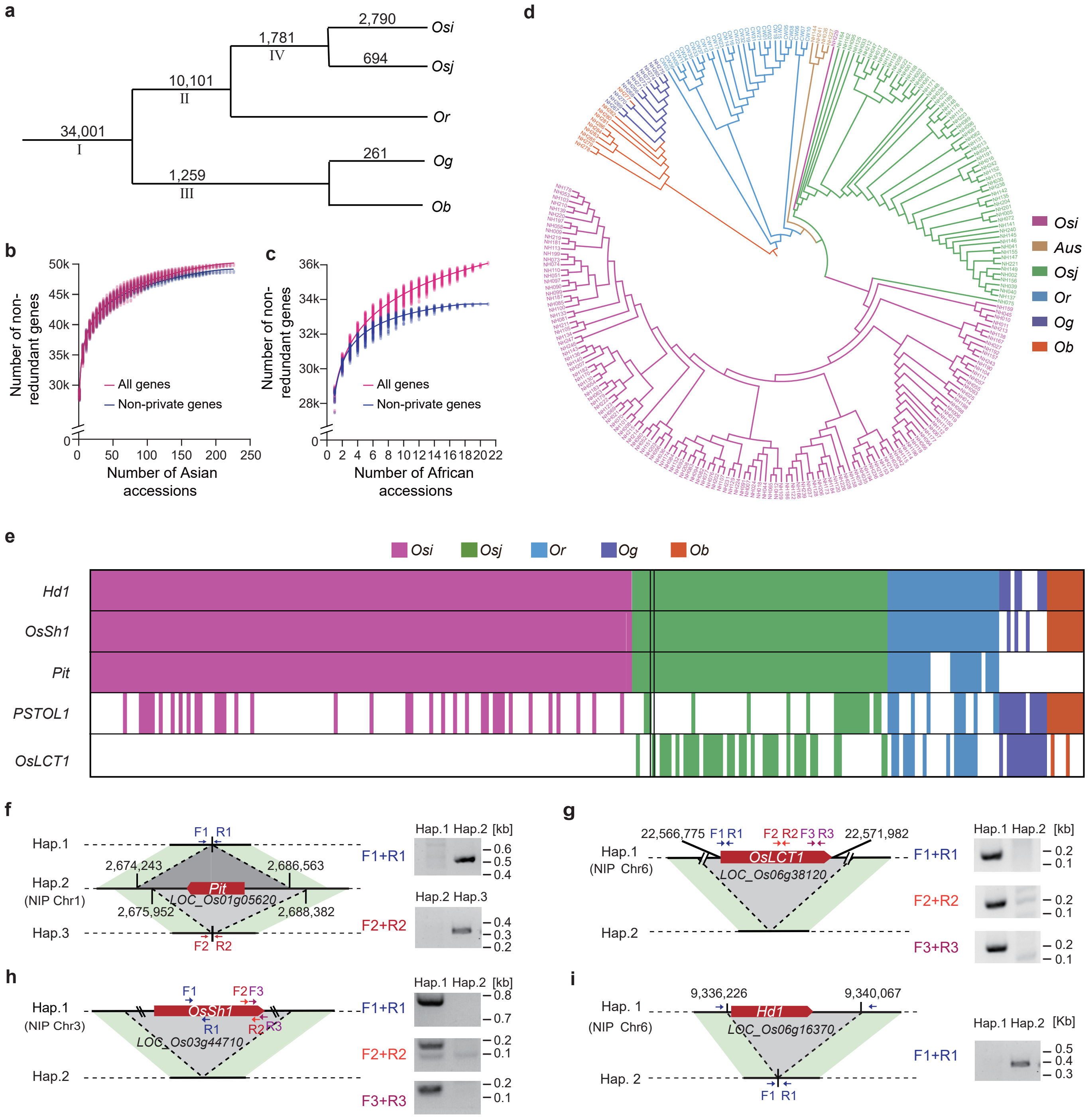

Fig. S2. Non-redundant genes integrated into the super pan-genome. **a** Distribution of non-redundant genes were inferred by the present pattern of genes in different accessions. Genes were grouped into four taxonomic levels, including I (Genes present in both *Or* and *Ob*), II (Genes present in both *Or* and *Os* except genes of level I), III (Genes present in both *Og* and *Ob* except genes of level I), IV (Genes present in both *Osi* and *Osj* except genes of level I and level II). 368 non-redundant genes were only shared by cultivated rice, and their origin time/species could not be determined. **b** Simulation of non-redundant gene number in Asian accessions based on 500 randomizations of rice genome orders. We used different sets of genes in the simulation: all genes, non-private genes (non-private genes are defined as non-redundant genes present in at least two accessions) in Asian rice. **c** Simulation of non-redundant gene number in African accessions based on 500 randomizations of rice genome orders. We used different sets of genes in the simulation: all genes, non-private genes (non-private genes are defined as non-redundant genes present in at least two accessions) in African rice. **d** Phylogeny of 251 accessions based on the presence/absence of non-redundant genes. Different colors indicate accessions in different sub-populations. **e** Presence/absence of important known genes including *Hd1* (functioning in rice heading date), *OsSh1* (loss function of *OsSh1* resulting in rice shattering-resistance), *Pit* (functioning in rice blast resistance), *PSTOL1* (presence of *PSTOL1* function promotes phosphorus-starvation tolerance in rice), and *OsLCT1* (functioning as a rice Cd transporter) across sub-populations. The vertical lines represent Nipponbare reference genome. Blank cell indicates absence of the gene. **f-i** Haplotypes exhibition and PCR validation of several selected and functionally characterized genes: *Pit* (**f**), *OsLCT1* (**g**), *Ossh1* (**h**), *Hd1* (**i**). Arrows indicate the positions of the primers. *Os*, *Osi*, *Osj*, *Or*, *Og* and *Ob* refer to *O. sativa*, *O. sativa indica*, *O. sativa japonica*, *O. rufipogon*, *O. glaberrima*, *O. barthii*, respectively .
