# Supplementary material for: A super pan-genomic landscape of rice
Source: Cell Res. 2022 Jul 12;32(10):878–96. doi: 10.1038/s41422-022-00685-z (PMC9525306; doi:10.1038/s41422-022-00685-z)
Supplement: Supplementary file 3 — Fig. S3 [file 41422_2022_685_MOESM3_ESM.pdf]

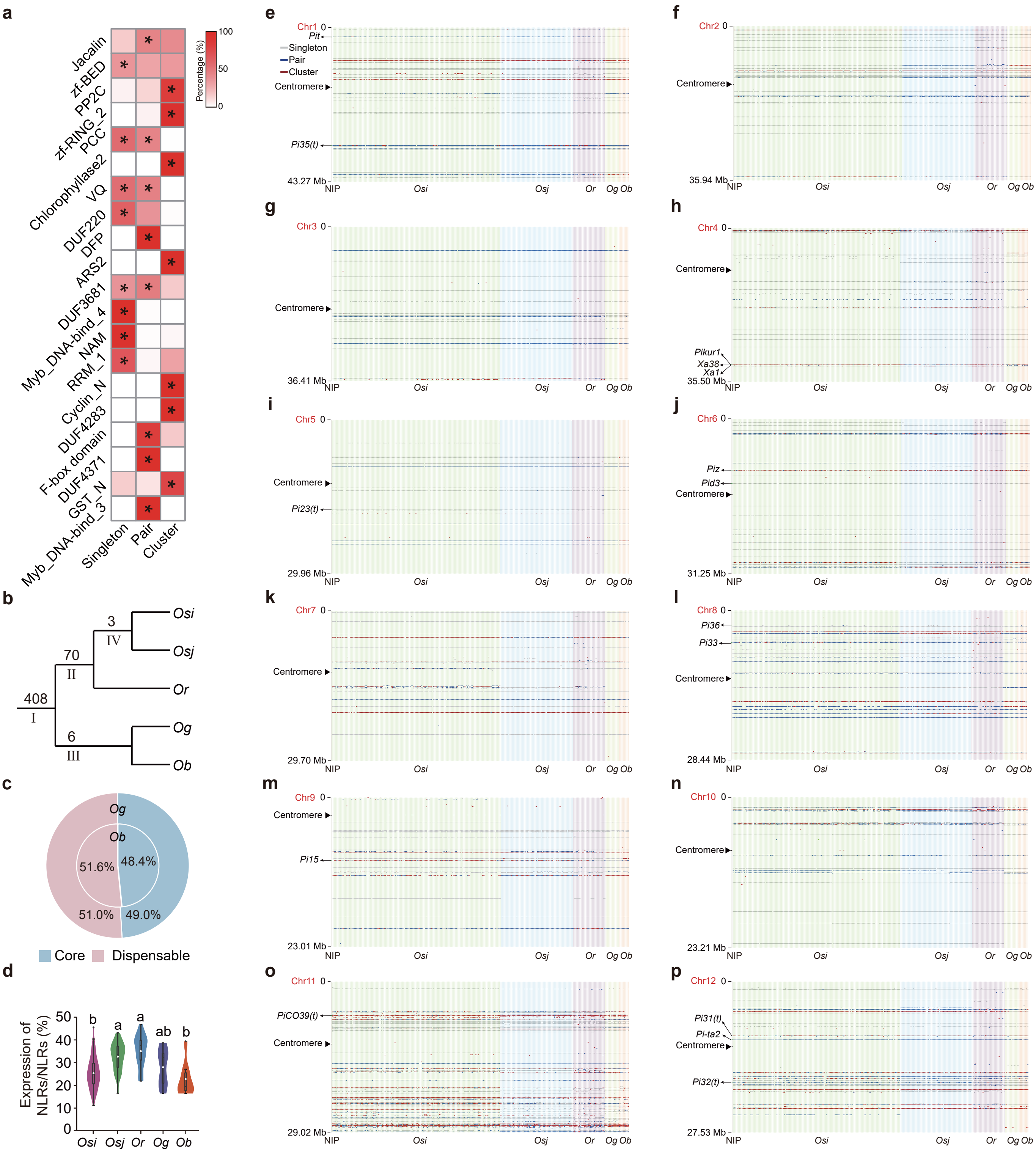

Fig. S3. Pan-NLRome analysis revealed NLRs diversity across different accessions. a The percentage of integrated domain in different types of NLRs (singleton, paired, and clustered NLRs). Enrichment was inferred by one-tailed Fisher's exact test with Benjamin-Hochberg adjustment. * adjusted $P < 0.05$. The integrated domain in the present figure are the same as those in Fig. 3e. **b** The distribution of NLRs non-redundant genes were inferred by the present pattern of genes in different accessions (same as Fig. S2a). **c** The percentage of core and dispensable non-redundant NLRs in *Ob* and *Og* populations. **d** Ratio of NLRs with FPKM >1 in different sub-populations. Mean is indicated by a white dot. The lowercase letters in the figure reflect the levels of statistical significance of the Kruskal–Wallis test with Bonferroni's multiple comparison post hoc test. **e-p** Genomic location of NLRs in the pan-genome graph. All the identified loci of NLRs were mapped to 12 chromosomes of the pan-genome graph. The NLRs in each locus are presented with colored blocks. The location of the known NLRs and their orthologous genes are indicated by collinearity loci aligning with the Nipponbare reference genome. Triangles indicate the centromere. Grey, blue, and red blocks indicate singleton, paired, and clustered NLRs, respectively. NIP indicates Nipponbare reference genome. *Osi*, *Osj*, *Or*, *Og*, and *Ob* refer to *O. sativa indica*, *O. sativa japonica*, *O. rufipogon*, *O. glaberrima*, and *O. barthii*, respectively.
