# Supplementary material for: A super pan-genomic landscape of rice
Source: Cell Res. 2022 Jul 12;32(10):878–96. doi: 10.1038/s41422-022-00685-z (PMC9525306; doi:10.1038/s41422-022-00685-z)
Supplement: Supplementary file 4 — Fig. S4 [file 41422_2022_685_MOESM4_ESM.pdf]

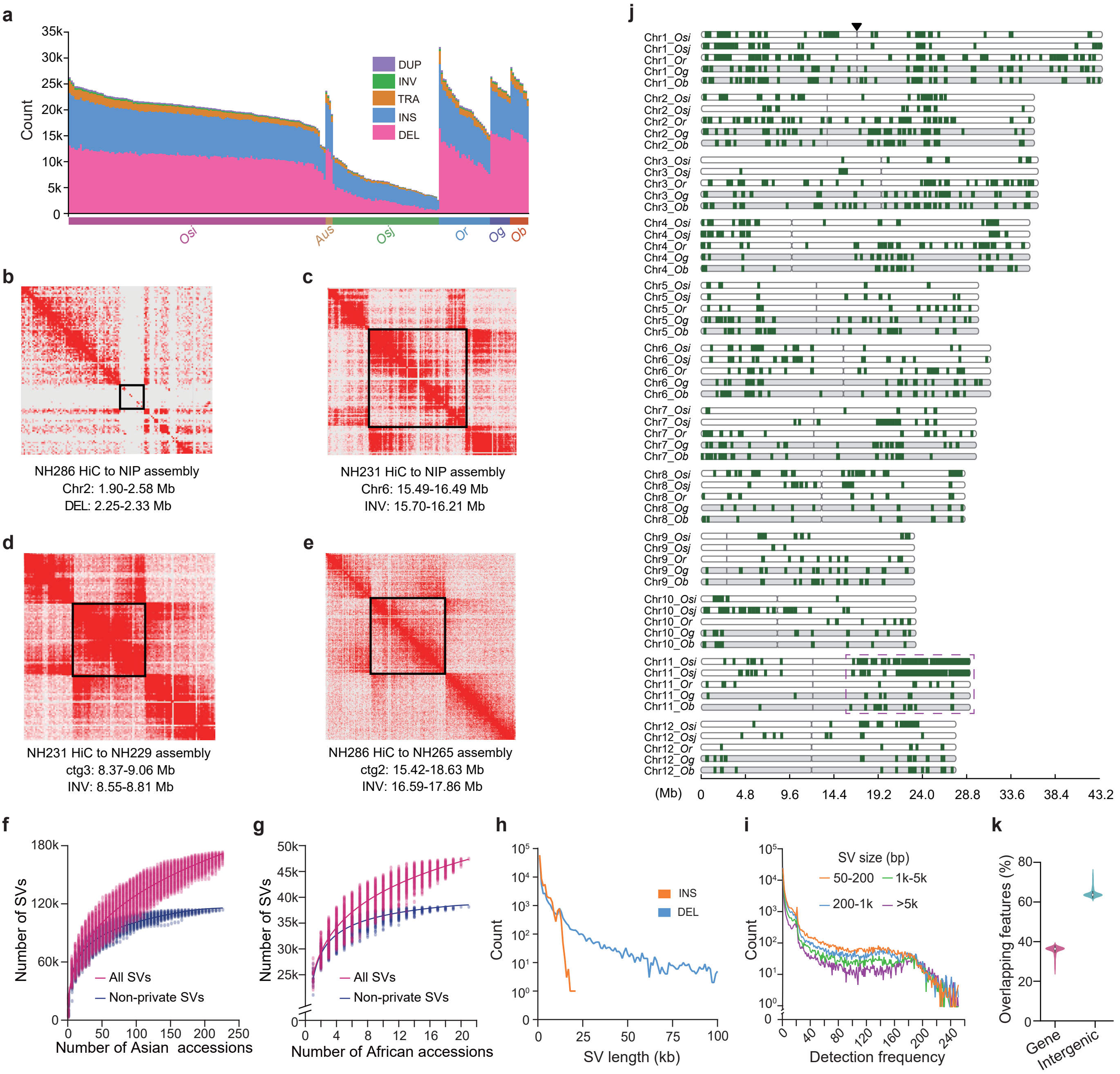

Fig. S4. Characteristics of pan-SV of 251 accessions. a Stacked histogram of SV numbers in 251 accessions. DUP, INV, TRA, INS and DEL refer to duplication, inversion, translocation, insertion and deletion, respectively. **b-e** Examples of manual validations of randomly selected large structural variations(SVs), based on mapping the Hi-C reads to the Nipponbare genome at 5 kb resolution. **f** Simulation of SV numbers in Asian rice accessions based on 500 randomizations of rice genome orders. We used different sets of SVs in the simulation: all SVs, non-private SVs (non-private SVs are defined as SVs present in at least two rice accessions) in Asian rice. **g** Simulation with SV numbers in African rice accessions based on 500 randomizations of rice genome orders. We used different sets of SVs in the simulation: all SVs, non-private SVs (non-private SVs are defined as SVs present in at least two rice accessions) in African rice. **h** Length distributions of DEL and INS in non-redundant SVs of 251 accessions, DEL and INS were removed if they were larger than 100kb and smaller than 50bp. **i** Detection frequencies for SVs with different lengths. **j** Chromosome distributions of hotspot SVs in *Osi*, *Osj*, *Or*, *Og* and *Ob*. The black triangle indicates centromere; the purple box indicates the hotspots on the long arm of Chromosome 11. **k** Features overlapping with SVs in each accession. *Osi*, *Aus*, *Osj*, *Or*, *Og*, and *Ob* refer to *O. sativa indica*, *O. sativa aus*, *O. sativa japonica*, *O. rufipogon*, *O. glaberrima*, and *O. barthii*, respectively.
