# Supplementary material for: A super pan-genomic landscape of rice
Source: Cell Res. 2022 Jul 12;32(10):878–96. doi: 10.1038/s41422-022-00685-z (PMC9525306; doi:10.1038/s41422-022-00685-z)
Supplement: Supplementary file 5 — Fig. S5 [file 41422_2022_685_MOESM5_ESM.pdf]

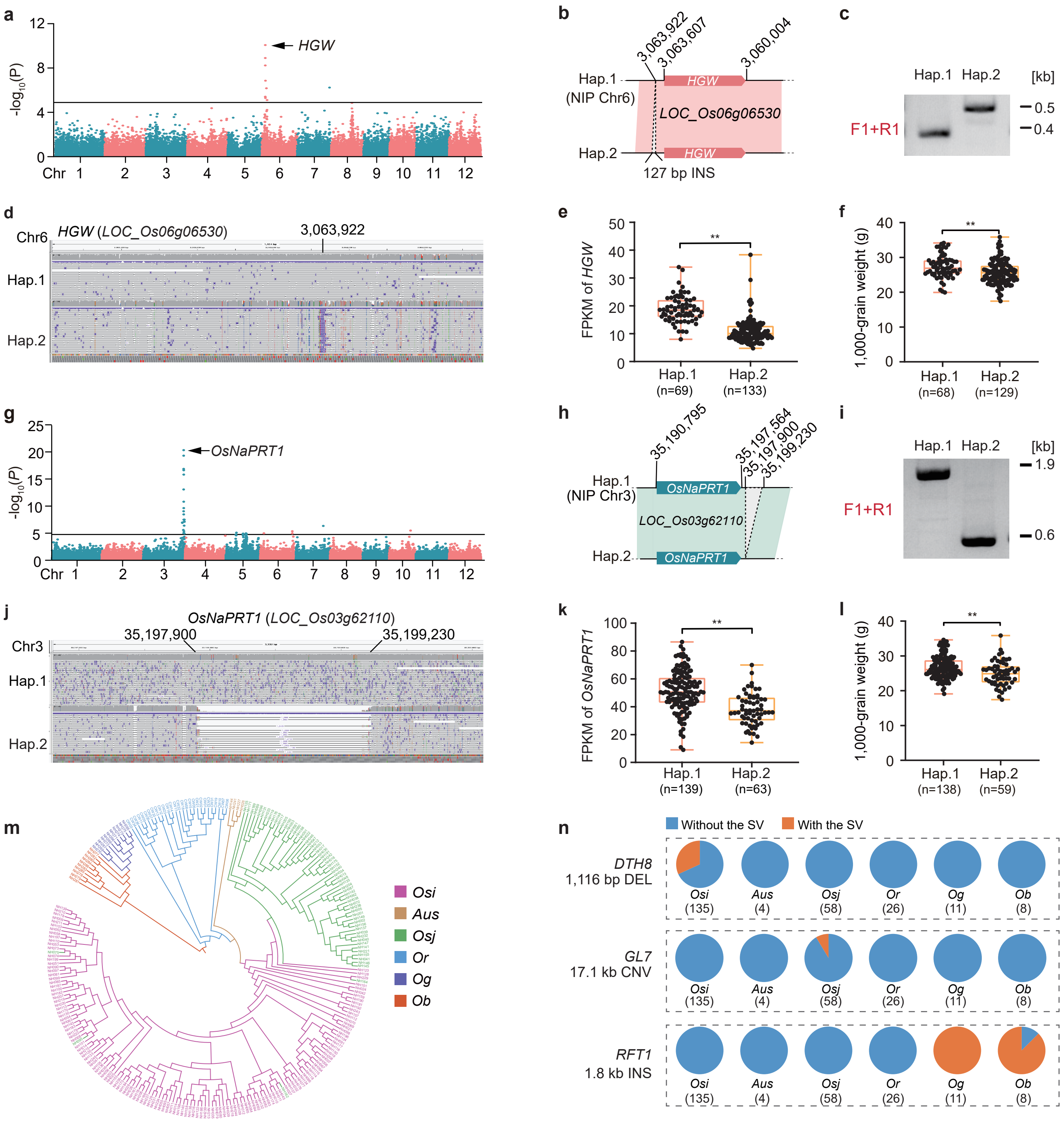


**Fig. S5. eQTL analysis using pan-SVs. a** Manhattan plot of the association between the pan-SV dataset and the expression levels of the *HGW* (*LOC_Os06g06530*) gene. **b** Haplotypes of the *HGW* gene in *Osi* and *Osj*. *Osi*, and *Osj* refer to *O. sativa indica* and *O. sativa japonica*, respectively. **c** Validation of the INS in *HGW* gene. **d** Breakpoint of the 127 bp INS in *HGW* gene in *Os* identified by mapping ONT reads to the Nipponbare reference genome. *Os* refer to *O. sativa*. **e** Expression of the *HGW* gene in accessions with or without the INS (against the Nipponbare reference genome). ** $P < 0.01$. Wilcoxon tests. **f** 1,000-grain weights of accessions with or without the INS in *HGW* gene. ** $P < 0.01$. Two-tailed t-tests. **g** Manhattan plot of the association between the pan-SV dataset and expression levels of *OsNaPRT1* (*LOC_Os03g62110*) gene. **h** Haplotypes of the *OsNaPRT1* gene in *Osi* and *Osj*. *Osi*, and *Osj* refer to *O. sativa indica* and *O. sativa japonica*, respectively. **i** Validation of the DEL in *OsNaPRT1* gene. **j** Breakpoint of the 1.3 kb DEL in *OsNaPRT1* gene in *Os* identified by mapping ONT reads to the Nipponbare reference genome. *Os* refer to *O. sativa*. **k** Expression of the *OsNaPRT1* gene in accessions with or without the DEL. ** $P < 0.01$. Two-tailed t-tests. **l** 1,000-grain weights of accessions with or without the DEL in *OsNaPRT1* gene. ** $P < 0.01$. Wilcoxon tests. **m** Phylogeny of 251 accessions based on SVs. Different colors indicate accessions in different sub-populations. **n** The distribution pattern of SVs of functional genes. *Osi*, *Aus*, *Osj*, *Or*, *Og*, and *Ob* refer to *O. sativa indica*, *O. sativa aus*, *O. sativa japonica*, *O. rufipogon*, *O. glaberrima*, and *O. barthii*, respectively.
