# Supplementary material for: A super pan-genomic landscape of rice
Source: Cell Res. 2022 Jul 12;32(10):878–96. doi: 10.1038/s41422-022-00685-z (PMC9525306; doi:10.1038/s41422-022-00685-z)
Supplement: Supplementary file 6 — Fig. S6 [file 41422_2022_685_MOESM6_ESM.pdf]

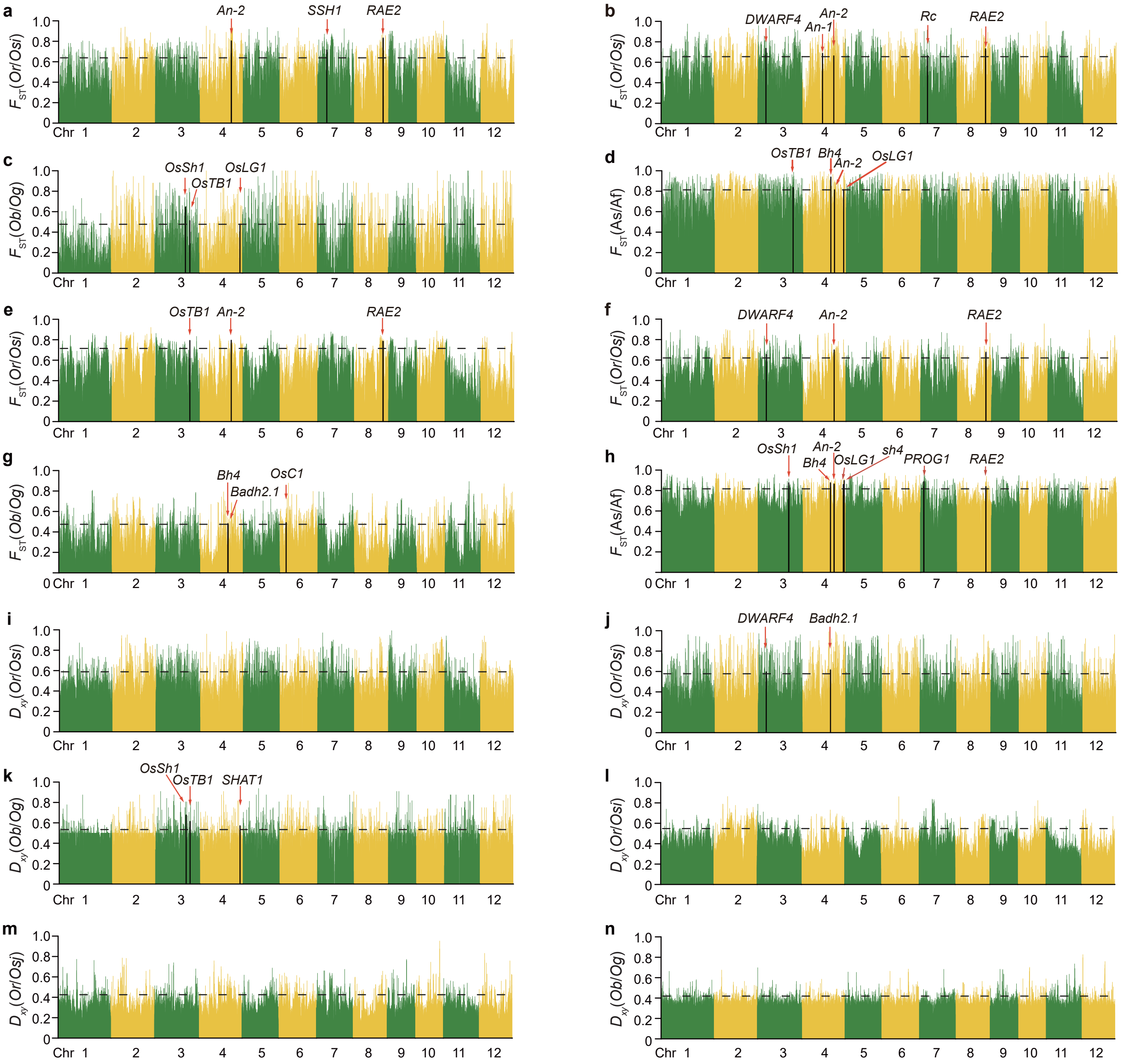

Fig. S6. Genetic variations contribute to domestication in Asian and African rice. **a-d** Divergent regions identified by F_{ST} using SVs: Manhattan plot of F_{ST} values between *Or* and *Osi* (**a**), F_{ST} values between *Or* and *Osj* (**b**), F_{ST} values between *Ob* and *Og* (**c**), and F_{ST} values between Asian and African rice (**d**). **e-h** Divergent regions identified by F_{ST} using SNPs: Manhattan plot of F_{ST} values between *Or* and *Osi* (**e**), between *Or* and *Osj* (**f**), between Ob and Og (**g**), and between Asian rice and African rice (**h**). **i-k** Divergent regions identified by D_{xy} using SVs: Manhattan plot of D_{xy} values between *Or* and *Osi* (**i**), between *Or* and *Osj* (**j**), and between *Ob* and *Og* (**k**). **l-n** Divergent regions identified by D_{xy} using SNPs: Manhattan plot of D_{xy} values between *Or* and *Osi* (**l**), between *Or* and *Osj* (**m**), and between *Ob* and *Og* (**n**). Manhattan plots are at 20 kb resolution; dashed lines indicate the top 5% of D_{xy} or F_{ST} values and black lines indicate known genes. *Osi*, *Osj*, *Or*, *Og*, and *Ob* refer to *O. sativa indica*, *O. sativa japonica*, *O. rufipogon*, *O. glaberrima*, and *O. barthii,* respectively.
