# Supplementary material for: A super pan-genomic landscape of rice
Source: Cell Res. 2022 Jul 12;32(10):878–96. doi: 10.1038/s41422-022-00685-z (PMC9525306; doi:10.1038/s41422-022-00685-z)
Supplement: Supplementary file 7 — Fig. S7 [file 41422_2022_685_MOESM7_ESM.pdf]

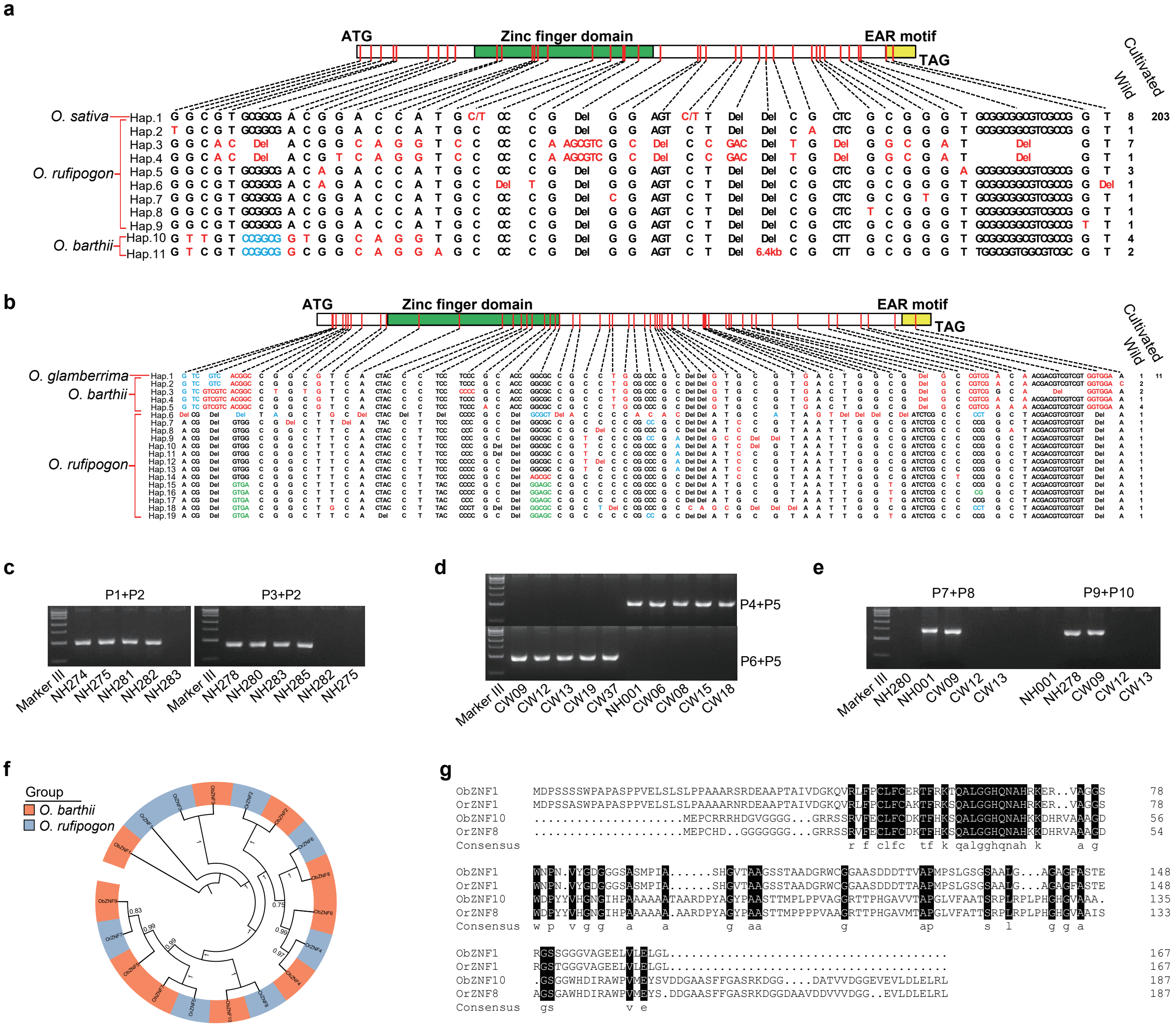

Fig. S7. Haplotype divergence of *RPAD* locus among sub-populations. a, b Haplotype analysis of *PROG1/PROG7* in *Os*, *Or*, *Ob*, and *Og*. Green and yellow boxes respectively indicate the zinc-finger domain and EAR motif of *PROG1*, while the red line shows the mutation site. **c** Validation of deletion in *Og* and *Ob* using primers P1, P2, and P3. **d** Validation of deletion in *Or* and *Os* using primers P4, P5, and P6. **e** Detection of variations in *PROG1* using primers P7 and P8 and detection of variations in *PROG7* using primers P9 and P10. The positions of primers P1-P10 are shown in Fig. 7a. **f** Phylogeny based on cDNA of *ObZNFs* and *OrZNFs*. **g** Alignment of protein sequences of *ObZNF1*, *OrZNF1*, *ObZNF10*, and *OrZNF8*. Identical residues are highlighted by black boxes. *Os*, *Osi*, *Osj*, *Or*, *Og*, and *Ob* refer to *O. sativa*, *O. sativa indica*, *O. sativa japonica*, *O. rufipogon*, *O. glaberrima*, and *O. barthii*, respectively.
